# A Redox‐Active Tetrathiafulvalene‐Based 3D Covalent Organic Framework with scu Topology for Controllable Charge Transport

**DOI:** 10.1002/smsc.202500489

**Published:** 2026-01-06

**Authors:** Tsukasa Irie, Jonas F. Pöhls, Saikat Das, Jin Sakai, Kohki Sasaki, Mika Nozaki, Yu Zhao, Luming Yang, Marina Bennati, Sourav Ghosh, Ranjit Thapa, Roland A. Fischer, R. Thomas Weitz, Qianrong Fang, Yuichi Negishi

**Affiliations:** ^1^ Institute of Multidisciplinary Research for Advanced Materials Tohoku University 2‐1‐1 Katahira Aoba‐ku Sendai 980‐8577 Japan; ^2^ First Institute of Physics Georg August University of Göttingen 37077 Göttingen Germany; ^3^ Department of Applied Chemistry Faculty of Science Tokyo University of Science Kagurazaka Shinjuku‐ku Tokyo 162‐8601 Japan; ^4^ Zhejiang Engineering Laboratory for Green Syntheses and Applications of Fluorine‐Containing Specialty Chemicals Institute of Advanced Fluorine‐Containing Materials Zhejiang Normal University Jinhua 321004 China; ^5^ Research Group EPR Spectroscopy Max Planck Institute for Multidisciplinary Sciences Am Fassberg 11 37077 Göttingen Germany; ^6^ Department of Physics SRM University–AP Amaravati Andhra Pradesh 522 240 India; ^7^ Centre for Computational and Integrative Sciences SRM University–AP Amaravati Andhra Pradesh 522 240 India; ^8^ TUM School of Natural Sciences Department of Chemistry Chair of Inorganic and Metal‐Organic Chemistry and Catalysis Research Center Technical University of Munich 85748 Garching Germany; ^9^ International Center for Advanced Study of Energy Conversion Göttingen ICASEC 37077 Göttingen Germany; ^10^ State Key Laboratory of Inorganic Synthesis and Preparative Chemistry Jilin University Changchun 130012 China

**Keywords:** covalent organic framework, electrical conductivity, iodine doping, redox‐active, tetrathiafulvalene

## Abstract

Unlike 2D frameworks where conductivity is largely confined to in‐plane transport, the **scu** topology offers 3D conduction pathways that enhance bulk charge mobility. When integrated with redox‐active species like tetrathiafulvalene (TTF), the **scu** architecture promotes electron transfer across the 3D network, enabling tunable conductivity. This article presents the construction of a 3‐periodic (4,8)‐c covalent organic framework (COF), TU‐48, adopting a twofold interpenetrated **scu** net, achieved through the integration of a tetratopic *D*_2h_‐symmetric rectangular TTF structural motif and an octatopic *D*_2h_‐symmetric quadrangular prism linker. TU‐48 exhibits high structural order, well‐defined porosity, and redox‐responsive electrochemical behavior. The high‐connectivity 3D COF configuration ensures effective access to TTF redox centers, enabling controlled iodine oxidation and resulting in electrical conductivities of 4.3 × 10^−6^ S cm^−1^ at 298 K and 1.8 × 10^−4^ S cm^−1^ at 393 K. By demonstrating how enhanced structural connectivity in TTF‐bridged 3D covalent lattices enables improved charge‐transport properties, this research fuels innovation in sustainable energy storage solutions and electronics.

## Graphical abstract

This article presents the designed construction of a high‐connectivity, electrochemically tunable 3D covalent organic framework (TU‐48) with a **scu‐c** topology, incorporating tetrathiafulvalene‐based building blocks to enable multidimensional charge transport and achieve enhanced electrical conductivity through redox modulation and iodine doping.

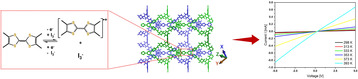

## 1 Introduction

Covalent organic frameworks (COFs) define a unique category of crystalline porous materials, precisely constructed through the covalent stitching of organic building blocks.^[^ ^1^, ^2^, ^3^, ^4^, ^5^, ^6^, ^7^, ^8^, ^9^, ^10^, ^11^, ^12^ ^]^ Noted for their extraordinary structural resilience and chemical flexibility, vast surface area, and regular porosity, COFs have piqued substantial interest for their applications in gas storage and separation,^[^ ^13^, ^14^, ^15^, ^16^ ^]^ heterogeneous catalysis,^[^ ^17^, ^18^, ^19^, ^20^ ^]^ optoelectronics,^[^ ^21^, ^22^, ^23^, ^24^ ^]^ and charge storage and transport.^[^ ^25^, ^26^, ^27^, ^28^ ^]^ Layered 2D COFs, bound by weak van der Waals interactions, frequently suffer from anisotropic charge transport, compromised stability, and interlayer slipping, which can hinder their functionality in energy and electronic applications.^[^ ^29^, ^30^ ^]^ Conversely, 3D COFs possess an extended, isotropic network of covalently connected organic motifs, which imparts greater structural stability, enhanced charge carrier dynamics, and more efficient molecular diffusion through interconnected pore channels.^[^ ^31^, ^32^ ^]^ The enhanced functionality of 3D COFs over 2D variants arises from their rigid and highly interconnected networks, which mitigate interlayer misalignment while promoting efficient electronic conduction, ion mobility, and structural resilience—crucial for charge storage, energy conversion, and catalytic applications. Notwithstanding these compelling advantages, the design and synthesis of highly connected 3D COFs remains a complex endeavor, as reconciling long‐range order—complicated by the high rotational flexibility of adjacent linkers—with high porosity and tailored functionality requires highly controlled synthesis and assembly techniques. While metal‐organic frameworks (MOFs) benefit from metal‐organic polyhedral nodes that enable coordination numbers of up to 24,^[^ ^33^ ^]^ 3D COF linkers still offer a relatively low number of extension points. Given these challenges, the exploration of new, highly connected 3D COFs featuring intricate topologies, tailored electronic characteristics, and robust chemical stability remains a critical pursuit in materials science.

On another note, tetrathiafulvalene (TTF) is a well‐known electron donor widely utilized in organic electronics for its high electron‐donating capability, which promotes efficient charge transfer, and its π‐conjugated structure, which supports charge delocalization and reinforces electronic coupling between adjacent units.^[^ ^34^, ^35^ ^]^ When TTF units are integrated into a 3D COF, they form an intrinsically conductive network, where charge carriers can migrate efficiently through a robust, covalently linked skeleton.^[^ ^36^ ^]^ This conductivity can be further modulated through charge transfer interactions, particularly when coupled with an appropriate electron acceptor unit such as iodine (I_2_). The interaction between I_2_ and TTF units induces partial oxidation of TTF, generating radical cations, which profoundly impact charge transport in 3D COFs. This oxidation leads to the formation of mixed‐valence charge‐transfer salts, facilitating continuous charge hopping across the extended network and significantly enhancing conductivity. The permanent porosity of COFs allows for efficient diffusion and uniform distribution of I_2_ molecules throughout the framework, ensuring homogeneous doping. Unlike conventional materials prone to charge trapping, the robust, crystalline 3D COF backbone provides superior stability against charge localization, preserving long‐range charge transport properties while minimizing charge scattering. The presence of conjugated TTF backbones in a highly connected 3D COF effectively transforms the material into a system of extended charge transport highways, where multiple redox‐active sites ensure that charge carriers can traverse the network seamlessly and efficiently.

**Scheme 1:**
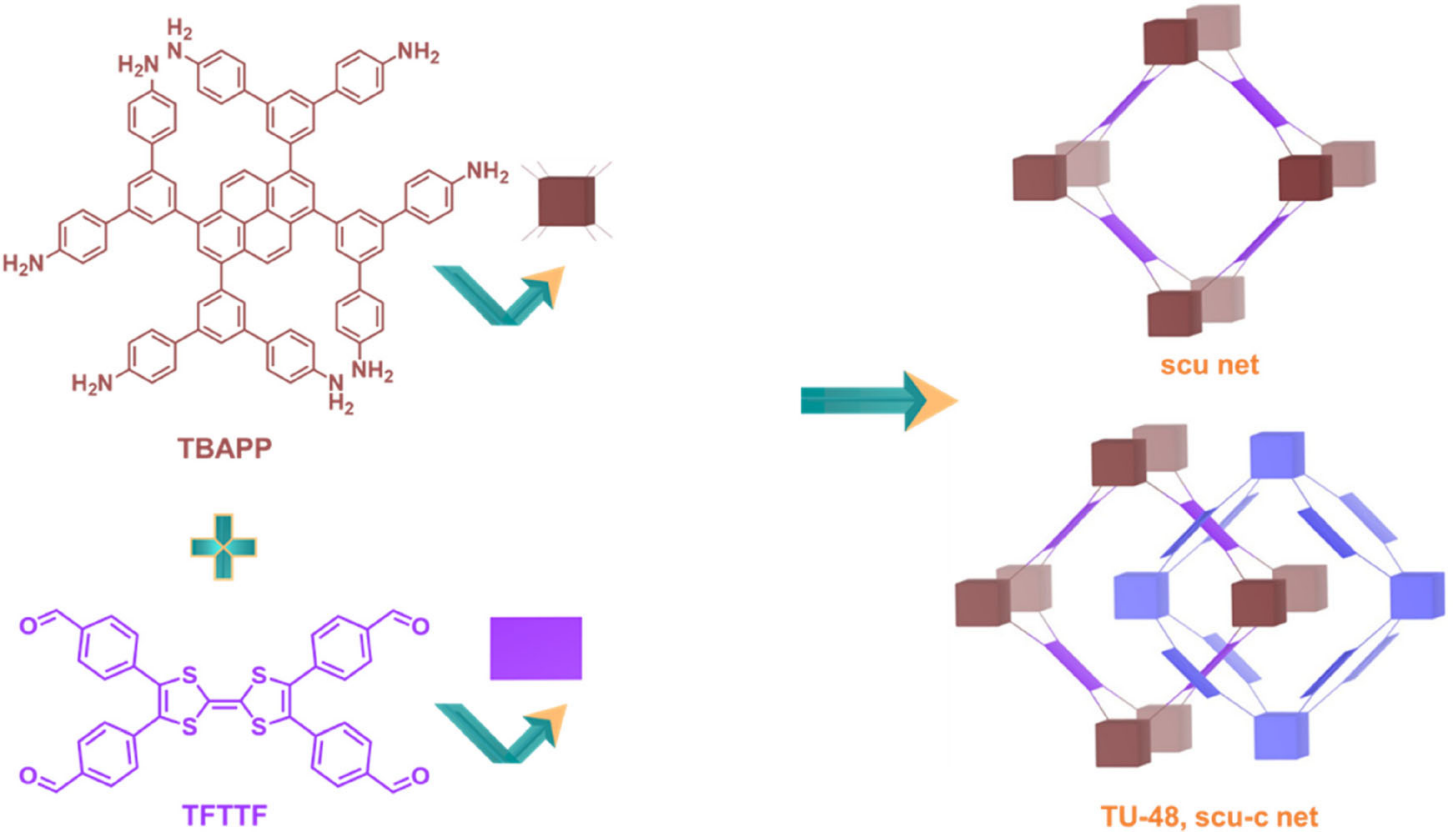
Structural design approach for 3D COF with **scu** Topology: Schiff‐base condensation between an 8‐connected 3D‐*D*_2h_ quadrangular prism monomer, TBAPP, and a 4‐connected 2D‐*D*_2h_ rectangular monomer, TFTTF, results in a 3D COF featuring a **scu‐c** net, classified under the **scu** topology.

In the quest for novel conductive COFs through the strategic assembly of highly intricate monomers possessing numerous extension sites, we herein reticulate an 8‐c building node, 1,3,6,8‐tetrakis‐3,5‐bis[(4‐amino)phenyl]phenylpyrene (TBAPP) with a 4‐c TTF derivative, 2,3,6,7‐tetra(4‐formylphenyl) tetrathiafulvalene (TFTTF), to develop a 3D COF, TU‐48 (TU = Tohoku University), characterized by a **scu‐c** net topology (**Scheme** **1**). The TTF units, with their inherent π‐conjugation and electron delocalization that aid charge mobility, along with the multidimensional charge pathways within the framework that allow for more efficient charge transport, demonstrate significantly improved conductivity of 1.8 × 10^−4^ S cm^−1^ at 393 K when doped with I_2_, highlighting TU‐48's suitability for electronic‐ and energy‐related applications. By leveraging the intrinsic redox activity of TTF units within a robust covalent lattice, TU‐48 establishes a blueprint for the rational design of highly conductive porous networks.

## 2 Results and Discussion

After optimizing various reaction parameters, TU‐48 was obtained by Schiff‐base polycondensation of TBAPP and TFTTF at a 1:2 molar ratio in *n*‐butanol, with 6 m aqueous acetic acid and aniline at 120 °C for 72 h, resulting in a high‐yield brown crystalline solid (refer to the Experimental Section for details). Fourier transform infrared (FT‐IR) and solid‐state ^13^C cross‐polarization magic angle spinning nuclear magnetic resonance (CP/MAS NMR) spectroscopies provided compelling evidence for the imine bond formation in the synthesized COF. The FT‐IR spectrum of TU‐48 displayed a prominent vibration band at 1620 cm^−1^, characteristic of C=N bonds, while the N—H stretching band at 3344 cm^−1^ (from TBAPP) and the C=O stretching band at 1695 cm^−1^ (from TFTTF) showed noticeable attenuation, supporting the occurrence of polycondensation (Figure S1, Supporting Information). To note, a weak residual C=O band at ≈1695–1700 cm^−1^ indicates reflects a small fraction of uncondensed terminal aldehyde groups, likely due to their limited accessibility within the interpenetrated 3D framework. The detection of a carbon resonance at 162 ppm, assigned to the C=N group, in the ^13^C CP/MAS NMR spectrum (Figure S2, Supporting Information) further validates the formation of an imine bond. Elemental analysis identified the atomic composition of TU‐48 as C_624_H_352_N_32_S_32_, with a high degree of consistency between experimental (C: 77.63; H: 4.46; N: 6.39; S: 11.52) and calculated (C: 80.41; H: 3.78; N: 4.81; S: 11:00) results, reinforcing the validity of COF construction. The morphology of TU‐48, as visualized through scanning electron microscopy (Figure S3, Supporting Information) and transmission electron microscopy (TEM) (Figure S4a, Supporting Information) appeared stick‐like, while high‐resolution TEM (HRTEM) images showcased well‐defined lattice fringes with consistent spacing (Figure 3c,d and S4b, Supporting Information). The lattice fringes observed in Figure 3c correspond to a d‐spacing of 0.86 nm, in excellent agreement with the (240) plane calculated from the COF structural model. TU‐48's thermal stability was assessed via thermogravimetric analysis in both nitrogen and air streams, confirming its resistance to temperatures up to 350 °C (Figure S5, Supporting Information). Additionally, TU‐48 retained its crystalline structure after 24‐h exposure to acidic (pH = 1), basic (pH = 14), and neutral (pH = 7) solutions at ambient temperature, as reflected in the powder X‐ray diffraction (PXRD) patterns (Figure S6, Supporting Information).

**Figure 1:**
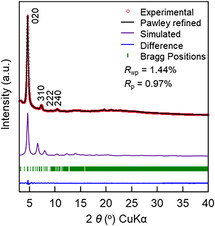
XRD diffractograms of TU‐48: observed diffraction pattern (red circles), Pawley‐refined fit (black curve), simulated pattern (purple curve) based on the **scu‐c** structural model, difference plot (blue curve) showing deviations between the experimental and refined patterns, and Bragg reflection positions (green ticks).

**Figure 2:**
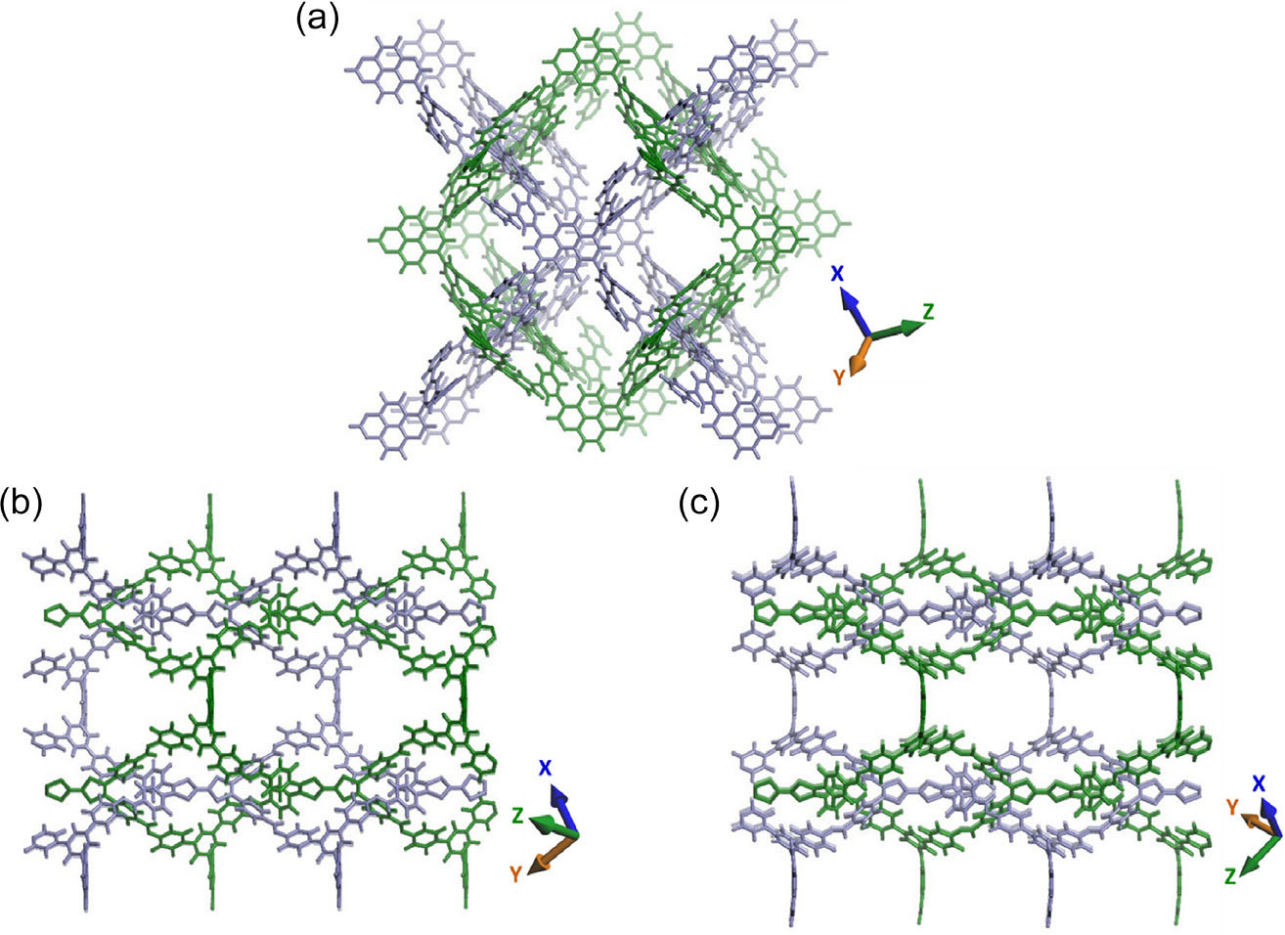
a–c) Visualizations of TU‐48's extended network along different axes.

Comprehensive assessment of the crystal structure and unit cell parameters of TU‐48 was achieved by correlating PXRD data with simulated models and structural refinement (Figure 1). The Forcite module in *Materials Studio* 7.0^[^ ^37^ ^]^ was employed for geometry optimization, leveraging force‐field‐based calculations to iteratively adjust the atomic positions and achieve a minimum‐energy configuration. This yielded the unit cell parameters of TU‐48 with a **scu‐c** net and *Fmm_2_* space group as *a* = 38.0258 Å, *b* = 37.8344 Å, *c* = 27.4576 Å, and *α* = *β* = *γ* = 90° (Table S4, Supporting Information). As evident in **Figure** **1**, the simulated diffraction pattern (purple trace) exhibits strong concordance with the experimental PXRD data (red dots). The experimentally recorded PXRD profile for TU‐48 featured characteristic Bragg reflections at 4.65°, 7.33°, 9.21°, and 10.44°, which correspond to the (020), (310), (222), and (240) crystal planes, respectively (Figure 1). Pawley refinement applied to the experimental diffraction data produced the refined unit‐cell parameters: *a* = 38.0404 Å, *b* = 37.8082 Å, *c* = 27.4506 Å, *α* = *β* = *γ* = 90°, with reliable agreement factors, *R*_p_ = 0.97%, *R*_wp_ = 1.44%. The Pawley‐refined PXRD pattern (black trace) corresponds closely with the experimental PXRD pattern (red dots), with only subtle differences noted in the difference profile (blue trace). A comprehensive structural assessment was undertaken to explore alternative topological assignments. Models evaluated included a noninterpenetrated **scu** topology in the *Cmm_2_* space group (Figure S15–S17,S19, Supporting Information), a twofold interpenetrated **scu** net in the *Cmc_2__1_* space group (Figure S21, Supporting Information), and a noninterpenetrated **csq** topology in the *P_6_/mmm* space group (Figure S23,S25, Supporting Information). In each case, the PXRD patterns simulated from the crystallographic models displayed clear deviations in peak positions relative to the experimental data (Figure S14,S18,S20,S22,S24, Supporting Information), indicating a mismatch between the proposed structures and the synthesized material. Through this process of systematic exclusion, the **scu‐c** topology in the *Fmm_2_* space group emerged as the only model that faithfully reproduces the experimental PXRD pattern, and is therefore assigned as the most consistent and structurally plausible model for TU‐48 (**Figure** **2**).

**Figure 3:**
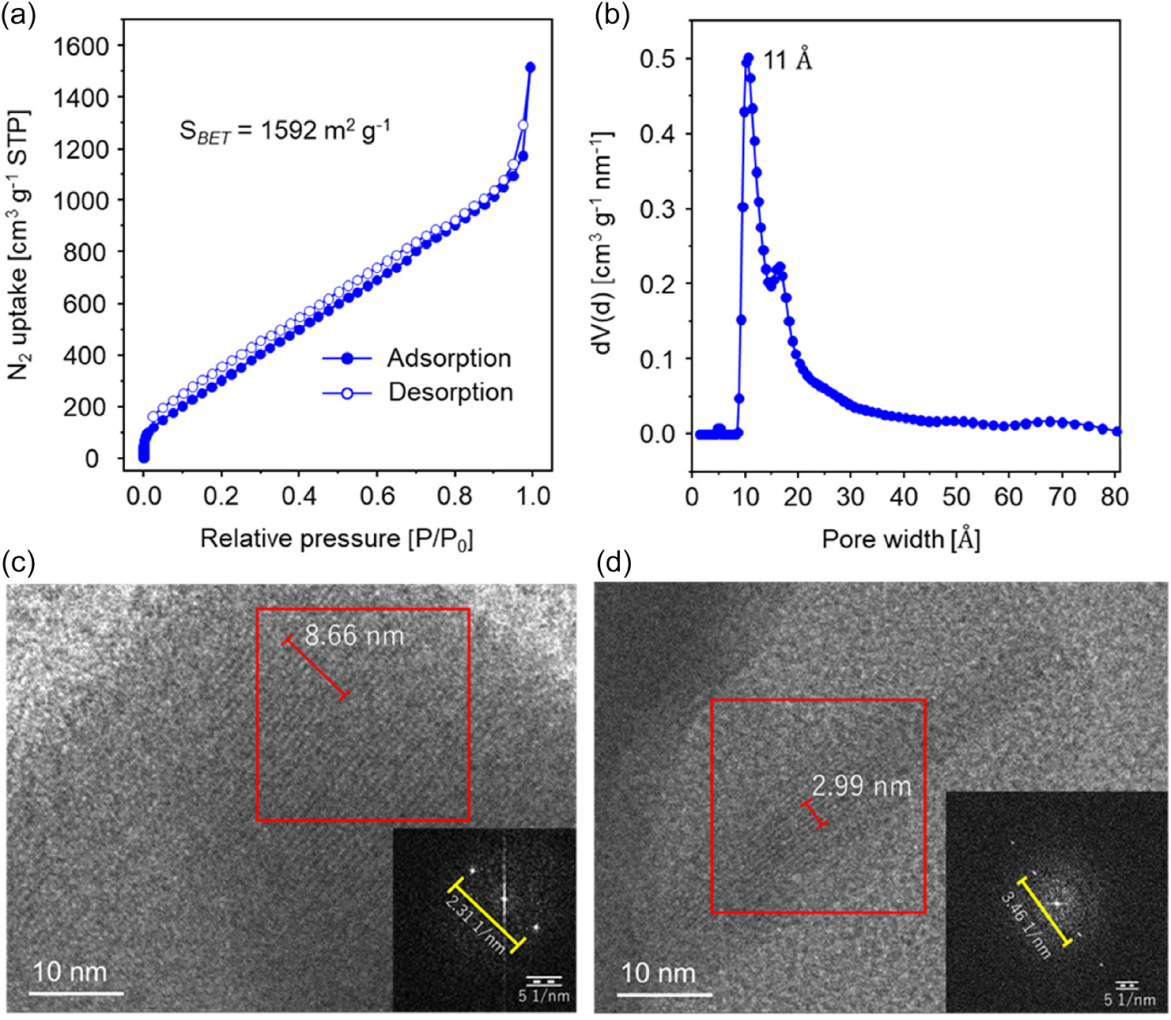
a) Nitrogen physisorption isotherms measured at 77 K and b) pore‐size distribution analyzed using NLDFT for TU‐48. c,d) HRTEM micrographs of TU‐48. Inset: fast Fourier transform (FFT) pattern obtained from the red‐framed area.

The porosity of TU‐48 was assessed through nitrogen adsorption–desorption isotherm measurements conducted at 77 K over a relative pressure (*P*/*P*_0_) range spanning from 0 to 1, subsequent to degassing at 120 °C for 8 h. The obtained isotherm exhibited a type I profile, distinguished by a sharp rise in nitrogen uptake at very low relative pressures, followed by the isotherm transitioning into a well‐defined plateau, which is a signature feature of microporous materials (**Figure** **3**a). Analysis of the adsorption data using the multipoint Brunauer–Emmett–Teller (BET) method derived a specific surface area of 1592 m^2^ g^−1^ (Figure S7, Supporting Information), whereas the BET surface identification protocol independently determined a statistically robust value of 1520 m^2^ g^−1^ (Figure S8, Supporting Information). The pore‐size distribution, generated from nonlocal density functional theory (NLDFT) analysis using the silica cylindrical pore model, exhibited a dominant peak appearing at 1.1 nm (Figure 3b), closely mirroring the simulated pore sizes of 1.1 and 1.3 nm.

**Figure 4:**
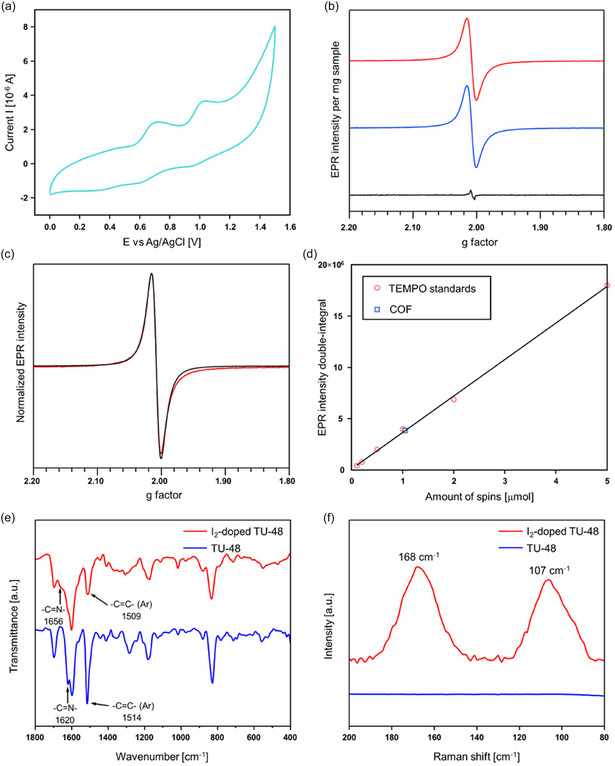
a) CV curve of TU‐48. b) Continuous‐wave X‐band EPR spectra of the pristine (black) and I_2_‐doped COF before (red) and after (blue) heating. EPR intensity is normalized to per milligram of material. c) Experimental (red) and simulated (black) EPR spectra of the I_2_‐doped COF before heating. Simulation was performed with Easyspin^[^ ^53^ ^]^ using *g*_iso_ = 2.0079 ± 0.0001, 2.6 mT Lorentzian, and 0.1 mT Gaussian line broadening for a spin‐1/2 system. d) Spin quantification of the I_2_‐doped COF based on doubly‐integrated EPR signal intensities. Blue square and red circles represent COF and TEMPO calibration standards, respectively. Solid black line is linear fit of the TEMPO data (*r* ^2^ > 0.99). Quantification was performed on 11.3 mg I_2_‐doped COF, which had 1.04 μmol radical. Calibration standards were toluene solutions of TEMPO with 1, 2, 5, 10, and 20 mM concentrations. e) FT‐IR and f) Raman spectra of TU‐48 and I_2_‐doped TU‐48.

As illustrated in **Figure** **4**a, TU‐48 displays a reversible cyclic voltammogram featuring two well‐defined redox processes at 0.708 and 1.034 V versus Ag/AgCl, confirming that the TTF moieties embedded within the COF framework remain electrochemically accessible and actively contribute to electron‐transfer processes. Upon exposure to I_2_ vapor, TU‐48 undergoes iodine doping, wherein I_2_ intercalates within the pores of the COF, as evidenced by a marked reduction in BET surface area from 1592 to 309 m^2^ g^−1^ (Figure S10, Supporting Information). The oxidation potential of TTF (first oxidation at 0.708 V vs. Ag/AgCl) aligns well with the reduction potential of I_2_ at 0.829 V vs. Ag/AgCl (0.784 V vs. SCE)^[^ ^38^, ^39^ ^]^ for I_3_^−^, allowing for efficient charge transfer. As a result, only the first redox process of TU‐48 is activated, selectively generating a partially oxidized COF with enhanced conductivity. Continuous‐wave X‐band electron paramagnetic resonance (EPR) spectroscopy of the pristine and I_2_‐doped COFs showed that the EPR signal became much stronger after I_2_ doping. The I_2_‐doped COF is described by isotropic *g* = 2.0079 ± 0.0001 (Figure 4b; calibrated against the Bruker Strong Pitch standard with *g* = 2.0028^[^ ^40^ ^]^), which is in good agreement with the values for the molecular derivatives of the TTF radical cation, ranging from 2.0073 to 2.0081.^[^ ^41^ ^]^ The peak‐to‐peak linewidth of 2.6 mT is on the same order as other reported I_2_‐doped TTF COFs (1.2–2.2 mT).^[^ ^42^, ^43^ ^]^ The line shape can be simulated with 96% Lorentzian and 4% Gaussian broadening (Figure 4c), suggesting homogeneous chemical environment of the oxidized TTF units. Calibration of the COF EPR intensity against 1–20 mM toluene solutions of TEMPO revealed radical concentration of 0.092 μmol mg^−1^ (Figure 4d), corresponding to about 0.8 radical per formula C_624_H_352_N_32_S_32_. Upon heating the I_2_‐doped COF at 393 K in air for 15 min, the EPR spectrum had negligible change in the *g*‐factor, line shape, and intensity (Figure 4b), reflecting thermal stability of the material. Power dependence of the EPR intensity was also checked to make sure that the EPR intensity varied linearly with square‐root of the microwave power above and below the selected power (Figure S27, Supporting Information). A clear shift in the stretching vibration peak of the C=N bond was observed in the FT‐IR spectral signature of the COF following I_2_ adsorption, signifying a pronounced interaction between the adsorbed iodine species and the imine linkages in the framework (Figure 4e). Additionally,

shifts in the C=C stretching vibrations of the benzene rings were also recorded after exposure to iodine vapor, implying a likely interaction between iodine molecules and the aromatic backbone of the COF. As illustrated in Figure 4f, the Raman spectrum of pristine TU‐48 exhibited no significant peaks. However, in the spectrum of I_2_‐doped TU‐48, two distinct peaks associated with the stretching band of I_5_^−^ and the symmetric stretching band of I_3_^−^ were observed at 168 and 107 cm^−1^, respectively.^[^ ^43^, ^44^, ^45^, ^46^, ^47^, ^48^, ^49^ ^]^ Moreover, the PXRD results demonstrated that TU‐48, following 48 h of I_2_ doping, sustained its crystalline structure and structural integrity (Figure S26, Supporting Information).

**Figure 5:**
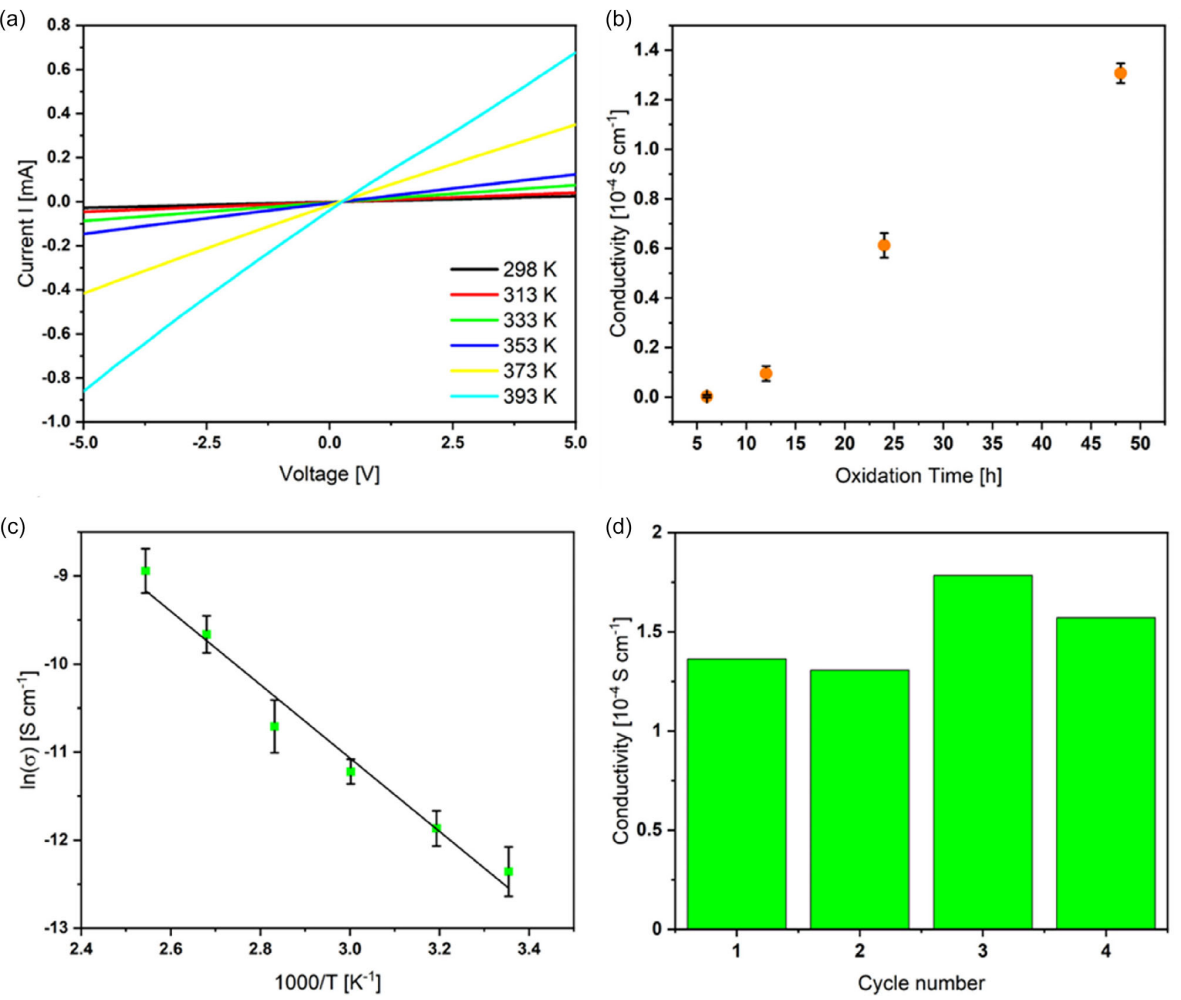
a) *I*–*V* response of TU‐48 under different temperatures following I_2_ oxidation for 48 h. b) Impact of I_2_ doping duration on the electrical conductivity of TU‐48 at 393 K. c) Arrhenius plot of TU‐48 after 48 h of I_2_ doping. d) Cycling stability of TU‐48 conductivities after 48 h I_2_ treatment at 393 K, conducted under ambient air. In Figure (b,c), the error bars represent the mean ± SD (*n* = 3).

Subsequently, an extensive evaluation of the electrical conductivities of iodine‐doped TU‐48 was carried out. The samples were molded into cylindrical pellets measuring 0.5 cm in diameter and 0.6 cm in thickness. The conductivity of the COF was measured using the two‐probe method, with the pellet set up as both the top and bottom electrode to secure optimal contact. Analysis of the *I*–*V* response of the doped COF samples across different temperatures revealed a steeper slope in the *I*–*V* curves at elevated temperatures, suggesting improved conductivity as charge transport became more efficient (**Figure** **5**a, S28–S30, Supporting Information). As reflected from the oxidation time‐dependent electrical conductivity plots (Figure 5b, S31–S35, Supporting Information), the conductivity of TU‐48 is adjustable with doping durations ranging from 6 to 48 h, which correlates with an increase in iodine uptake from 47.8 wt% at 6 h to 214.0 wt% at 48 h. Given that the redox interaction between TTF and I_2_ is central to the doping mechanism, it is pertinent to highlight the increasing I_2_/TTFTTF molar ratio—from 2.09 to 9.37 mol mol^−1^—as the doping time is extended (Table S1, Supporting Information). As detailed in Table S2, Supporting Information, TU‐48 initially exhibits a conductivity of 5.1 × 10^−9^ S cm^−1^ at 298 K after 6 h of doping, which rises to 4.3 × 10^−6^ S cm^−1^ at 298 K with a prolonged doping duration of 48 h. Increasing the temperature to 393 K resulted in a dramatic conductivity improvement to 1.8 × 10^−4^ S cm^−1^. The linear fit of **ln *σ*** against 1/T supports an Arrhenius‐type thermally activated transport mechanism, yielding an activation energy (***E***_**a**_) of 0.36 eV (Figure 5c). Remarkably, the conductivity of TU‐48 after 48 h of I_2_ doping surpasses that of 2D TTF‐COF doped for 48 h (1.8 × 10^−6^ S cm^−1^).^[^ ^43^ ^]^ This is mainly due to the multidimensional charge transport pathways, better charge carrier delocalization, more effective iodine doping, fewer charge‐trapping sites, and enhanced orbital overlap in 3D COFs. In addition, doped TU‐48 exhibits a higher conductivity than doped TTF‐based HOF‐110 (6.0 × 10^−7^ S cm^−1^)^[^ ^50^ ^]^ and TTF‐based MOFs viz. In(Me_2_NH_2_)(TTFTB) (1.68 × 10^−7^ S cm^−1^)^[^ ^51^ ^]^ and (TCNE)_0.81_@Zn_2_TTFTB (1.17 × 10^−5^ S cm^−1^).^[^ ^52^ ^]^ Table S3, Supporting Information summarizes the electrical conductivities of representative 2D and 3D COFs and MOFs following post‐treatment (e.g., iodine doping, redox activation) in comparison with TU‐48. Besides, TU‐48 maintained a relatively stable electrical conductivity after 48 h of I_2_ exposure at 393 K, demonstrating steady performance across four successive tests with no major electroactive deterioration (Figure 5d).

## 3 Conclusion

We successfully designed and synthesized a robust electrochemically tunable (4,8)‐c 3D framework with **scu‐c** topology through the assembly of TTF‐based tetratopic *D*_2h_‐symmetric rectangular and octatopic *D*_2h_‐symmetric quadrangular prism building units displaying high structural order, well‐defined porosity, and tunable electrochemical functionality. By incorporating TTF units into the 3D COF network, TU‐48 achieves intrinsic charge mobility through π‐conjugation, electron delocalization, and multidimensional charge pathways. When coupled with I_2_ doping, the charge transfer interactions further enhance electrical conductivity by creating TTF‐I_3_^−^ (triiodide) and TTF‐I_5_^−^ (pentaiodide) charge‐transfer complexes, leading to an electrical conductivity of 4.3 × 10^−6^ S cm^−1^ at 298 K and 1.8 × 10^−4^ S cm^−1^ at 393 K. This study highlights how precise structural design can optimize charge transport pathways in 3D COFs. The ability to fine‐tune redox behavior and charge transport by modulating topological connectivity opens new avenues for the development of organic conductive materials for applications in batteries, supercapacitors, and organic electronics.

## 4 Experimental Section

4.1

4.1.1

##### Synthesis of TU‐48

A Pyrex tube with 8 mm inner diameter ×10 mm outer diameter was charged with TBAPP (24.7 mg, 0.02 mmol), TFTTF (24.8 mg, 0.04 mmol), and 1.0 mL of anhydrous *n*‐butanol. The mixture was ultrasonic treated for about 15 min. Next, 0.2 mL of 6 M aqueous acetic acid was introduced, followed by an additional 15‐min sonication. Afterward, 0.1 mL of aniline was incorporated, and the tube was subjected to another 15‐min sonication to achieve uniform dispersion. The prepared tube was flash frozen at 77 K in a liquid nitrogen bath, evacuated to an internal pressure below 0.2 mbar, and flame‐sealed. The length of the sealed tube was reduced to ≈9 cm. Once the tube returned to ambient temperature, it was heated at 120 °C for 3 days. Upon completion, the tube was retrieved and allowed to cool to room temperature. The resulting precipitate was collected via centrifugation and extensively washed with THF until the eluate turned colorless. A Soxhlet extraction was performed using THF for 24 h for further purification. The air‐dried solid was then subjected to vacuum drying at room temperature for 4 h, followed by overnight degassing at 100 °C under vacuum, yielding activated TU‐48 as a brown solid with an 82% yield. Anal. Calcd. for C_624_H_352_N_32_S_32_: C: 80.41; H: 3.78; N: 4.81; S: 11:00. Found: C: 77.63; H: 4.46; N: 6.39; S: 11.52.

##### Doping TU‐48 with Iodine

The degassed COF sample (≈ 42.0 mg) in a 4.0 mL open vial was positioned inside a sealed 25.0 mL vial with 1.5 g of iodine beads and maintained at 75 °C on a hot plate for a predetermined duration. Following cooling to ambient temperature, the COF‐loaded small vial was removed, and aliquots of the doped sample were collected for conductivity analysis.

##### Cyclic Voltammetry (CV)

For electrode fabrication, the as‐prepared COF (6.0 mg) and carbon black (4.0 mg) were thoroughly ground in a mortar and pestle until a uniform fine powder was achieved. This mixture was then dispersed in water (0.25 mL), ethanol (0.25 mL), and Nafion solution (50.0 μL), followed by sonication for 2 h to ensure homogeneous dispersion. The resulting ink‐like suspension was drop‐cast onto a carbon fiber substrate, which was dried prior to use. CV was conducted with a CHI 760 E electrochemical workstation in a standard three‐electrode configuration, comprising a carbon working electrode, platinum wire counter electrode, and an Ag/AgCl reference electrode. The active electrode area had a diameter of 4 mm. Measurements were recorded at a scan rate of 0.02 V s^−1^, with a sampling interval of 0.001 V, across a 0–1.5 V potential window, using 0.1 m NBu_4_PF_6_ in CH_2_Cl_2_ as the electrolyte solution.

##### Continuous‐Wave X‐Band EPR Spectroscopy

Continuous‐wave X‐band EPR spectra were acquired on a Bruker Elexsys E500T spectrometer (microwave frequency ≈ 9.86 GHz) equipped with a high‐Q cylindrical resonator. COF and TEMPO samples were prepared to have the same height inside the EPR sample tubes. The pristine and I_2_‐doped COF samples contained 5.7 and 11.3 mg materials, respectively. Spectra were collected with 1 mW microwave power, 100 kHz modulation frequency, 0.2 G modulation amplitude, 24.7 ms conversion time, 4096 points, and 10 averages. *g*‐factor calibration was performed by B_0_ magnetic field correction based on the Bruker Strong Pitch reference sample. The COF sample was first calibrated against 10 mM TEMPO solution in toluene, which was further calibrated against the Strong Pitch reference. The calibrated *g*_iso_ for the TEMPO toluene solution was 2.0061 ± 0.0001.

##### Conductivity Measurements

The COF's conductivity was examined using the two‐probe technique, where 42.0 mg of COF was compacted into cylindrical pellets (0.5 cm in diameter, 0.6 cm in thickness) using a custom‐built pellet press at 3.0 MPa. The pellet was configured to serve as both the top and bottom electrode, ensuring complete contact. These contacts were connected to a Keithley Model 2450 Source Meter to record current–voltage (*I*–*V*) data in the −5–5 V range, with temperature‐dependen*t* testing from 298 to 393 K.

##### Statistical Analysis

All statistical data presented in Figure 5b,c and S31–S35, Supporting Information, are reported as mean ± standard deviation (SD), depicted as error bars. The sample size (*n*) for each measurement is specified in the corresponding figure captions (e.g., *n* = 3). All statistical analyses were performed using OriginPro software.

## Supporting Information

Supporting Information is available from the Wiley Online Library or from the author.

## Conflict of Interest

The authors declare no conflict of interest.

## Supporting information

Supplementary Material

## Data Availability

The data that support the findings of this study are available from the corresponding author upon reasonable request.
