# Supplementary material for: A Redox‐Active Tetrathiafulvalene‐Based 3D Covalent Organic Framework with scu Topology for Controllable Charge Transport
Source: Small Sci. 2026 Jan 6;6(1):e202500489. doi: 10.1002/smsc.202500489 (PMC12774326; doi:10.1002/smsc.202500489)
Supplement: Supplementary file 1 — Supplementary Material [file SMSC-6-e202500489-s001.pdf]

Supporting Information

A Redox-Active Tetrathiafulvalene-Based Three-Dimensional Covalent Organic Framework with scu Topology for Controllable Charge Transport

Tsukasa Irie,[a,k] Jonas F Pöhls,[b,k] Saikat Das,*[a] Jin Sakai,[c] Kohki Sasaki,[a] Mika Nozaki,[a] Yu Zhao,[d] Luming Yang,[e] Marina Bennati,[e] Sourav Ghosh,[f] Ranjit Thapa,[f,g] Roland A. Fischer,[h] R. Thomas Weitz,*[b,i] Qianrong Fang,*[j] and Yuichi Negishi*[a]

[a]Institute of Multidisciplinary Research for Advanced Materials, Tohoku University, 2-1-1 Katahira, Aoba-ku, Sendai 980-8577, Japan

[b]First Institute of Physics, Georg August University of Göttingen, 37077 Göttingen, Germany

[c]Department of Applied Chemistry, Faculty of Science, Tokyo University of Science, Kagurazaka, Shinjuku-ku, Tokyo 162-8601, Japan

[d]Zhejiang Engineering Laboratory for Green Syntheses and Applications of Fluorine-Containing Specialty Chemicals, Institute of Advanced Fluorine-Containing Materials, Zhejiang Normal University, 321004 Jinhua, China

[e]Research Group EPR Spectroscopy, Max Planck Institute for Multidisciplinary Sciences, Am Fassberg 11, Göttingen 37077, Germany

[f]Department of Physics, SRM University–AP, Amaravati 522 240, Andhra Pradesh, India

[g]Centre for Computational and Integrative Sciences, SRM University–AP, Amaravati 522 240, Andhra Pradesh, India

[h]TUM School of Natural Sciences, Department of Chemistry, Chair of Inorganic and Metal-Organic Chemistry and Catalysis Research Center, Technical University of Munich, Garching, Germany

[i]International Center for Advanced Study of Energy Conversion, Göttingen ICASEC, Göttingen 37077, Germany

[j]State Key Laboratory of Inorganic Synthesis and Preparative Chemistry, Jilin University, Changchun 130012, China

[k]These authors contributed equally.

*Email: das.saikat.c4@tohoku.ac.jp; thomas.weitz@uni-goettingen.de; qrfang@jlu.edu.cn; yuichi.negishi.a8@tohoku.ac.jp

List of Contents

1. Materials and methods S3

2. Fourier-Transform Infrared (FT-IR) Spectroscopy S5

3. Solid-state ^{13}C CP-MAS Nuclear Magnetic Resonance Spectroscopy S6

4. Scanning electron microscopy (SEM) and Transmission electron microscopy (TEM) characterization S7

5. Thermogravimetric analysis (TGA) S8

6. Chemical stability analysis S9

7. Nitrogen sorption S10

8. Diffuse reflectance spectroscopy (DRS) S16

9. Energy values of HOCO and LUCO of the TU-48 COF from band structure calculations S17

10. Structural modeling and X-ray diffraction (XRD) analyses S19

11. Iodine doping S25

12. Continuous-wave X-band electron paramagnetic resonance (EPR) spectroscopy S26

13. Electrical conductivities of I_2-doped TU-48 S27

14. Unit cell information and fractional atomic coordinates S35

15. Supplementary references S39

1. Materials and methods

1.1. Reagents. All chemicals and solvents were of analytical grade and, unless stated explicitly, were used as received without additional purification. Anhydrous *n*-butanol, acetic acid, tetrahydrofuran (THF), iodine beads, and Nafion solution were procured from FUJIFILM Wako Pure Chemical Corporation. Aniline was procured from Tokyo Chemical Industry Co., Ltd. Ethanol and dichloromethane (CH_2Cl_2) were procured from Kanto Chemical Co., Inc. Carbon black was procured from Fuel Cell Earth. Carbon fiber substrate was procured from SGL carbon.

1.2. Instrumentation.

Powder X-ray diffraction (PXRD): A Rigaku RINT-2000 X-ray diffractometer, equipped with CuKα radiation (λ = 1.5418 Å) at 40 kV and 40 mA, was used to collect PXRD data within a 2θ range of 3°–40°, employing a step size of 0.02° and a scan speed of 0.2° per min.

Fourier transform infrared (FT-IR) spectroscopy: Attenuated total reflectance Fourier transform infrared spectroscopy (ATR-FTIR) spectral measurements were carried out on a JASCO FT/IR-4600 FT-IR spectrometer across a wavenumber range of 4000–400 cm^{-1}.

Solid-state ^{13}C cross-polarization magic-angle-spinning (CP-MAS) NMR spectroscopy: Solid-state ^{13}C CP-MAS NMR spectra were acquired on a JEOL ECZL-400 NMR spectrometer operating at a ^{13}C resonance frequency of 100.52 MHz and spinning rate of 15 kHz.

Scanning electron microscopy (SEM): A JEOL JSM-7000FSHL field emission scanning electron microscope, operated at 3 kV, was used to acquire SEM images, and the samples were gold sputter-coated beforehand using a Sanyu Electron SC-701MkⅡ ADVANCE sputterer to reduce charging.

High-resolution transmission electron microscopy (HRTEM): A JEOL JEM-2100F microscope, operated at 80 kV, was used to acquire HRTEM images and conduct fast Fourier transform (FFT) analysis.

Thermogravimetric analysis (TGA): TGA measurements were performed using a Bruker TG-DTA2010SA instrument, heating the sample in a nitrogen/air atmosphere from room temperature to 800 °C at 10 °C min^{-1}, with an N_2/air flow rate of 50 mL min^{-1}.

Nitrogen sorption: A Quantachrome Autosorb iQ gas sorption analyzer was employed to measure nitrogen adsorption-desorption isotherms at 77 K. Prior to analysis, the sample underwent activation at 120 °C for 8 hours under dynamic vaccum. The specific surface area was estimated through

multipoint BET analysis, and the pore size distribution was derived from NLDFT calculations based on the N_2@77 K on silica cylindrical pore model.

Raman spectroscopy: Raman spectra were recorded using a Horiba Jobin Yvon LabRAM HR-800 Raman spectrometer with a 532 nm excitation wavelength.

Elemental analyses: The elemental composition of the COF was determined with an Elementar vario EL cube elemental analyzer.

1.3. Synthesis of building blocks. 1,3,6,8-tetrakis-3,5-bis[(4-amino)phenyl]phenylpyrene (TBAPP)[1] and 2,3,6,7-tetra(4-formylphenyl) tetrathiafulvalene (TFTTF)[2] were synthesized according to previously reported methods.

1.4. Computational details. All structural relaxations, density of states, and band-structure calculations were carried out using the open-source Quantum ESPRESSO package.[3-5] Exchange-correlation effects were treated with the Perdew-Burke-Ernzerhof (PBE) functional within the Generalized Gradient Approximation (GGA).[6] Long-range dispersion interactions were incorporated through the DFT-D3 van der Waals correction with damping to accurately capture intermolecular forces. For geometry optimization, the electronic and ionic convergence thresholds were set to 7.35 x 10^{-7} Ry (~10^{-5} eV) and 1 x 10^{-4} Ry (~10^{-3} eV), respectively. A force convergence criterion of 7.78 x 10^{-4} Ry/Bohr (0.02 eV/Å) was applied throughout. Brillouin-zone sampling employed a 1 x 1 x 1 Monkhorst–Pack grid for structural relaxation, while a denser 3 x 3 x 1 grid was used for the DOS calculations.[7] Gaussian smearing with a width of 0.01 Ry was introduced to facilitate electronic convergence.

2. Fourier-Transform Infrared (FT-IR) Spectroscopy

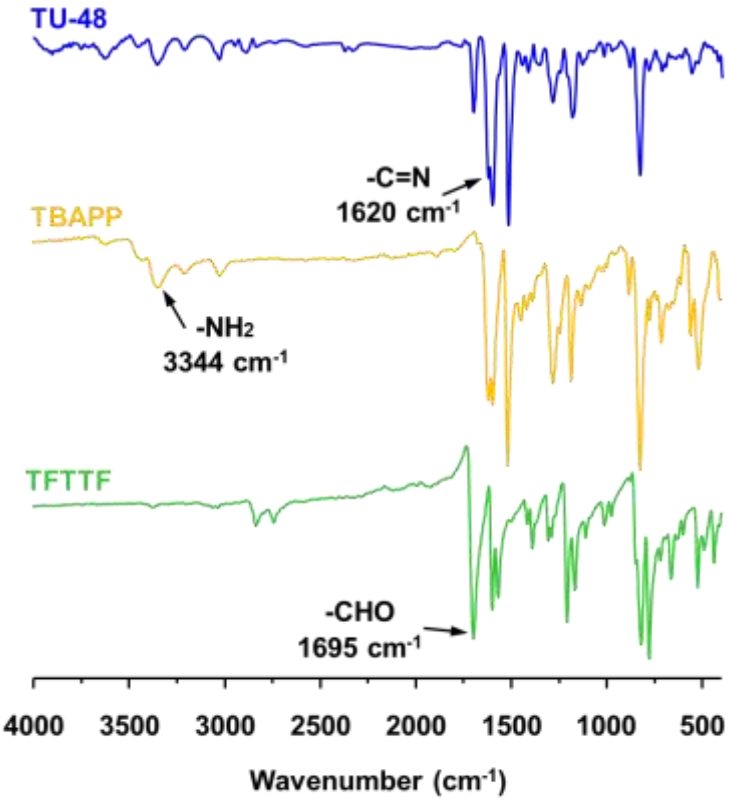

Figure S1. FT-IR spectra of TU-48 (blue), TBAPP (yellow), and TFTTF (green).

3. Solid-state ^{13}C CP-MAS Nuclear Magnetic Resonance (NMR) Spectroscopy

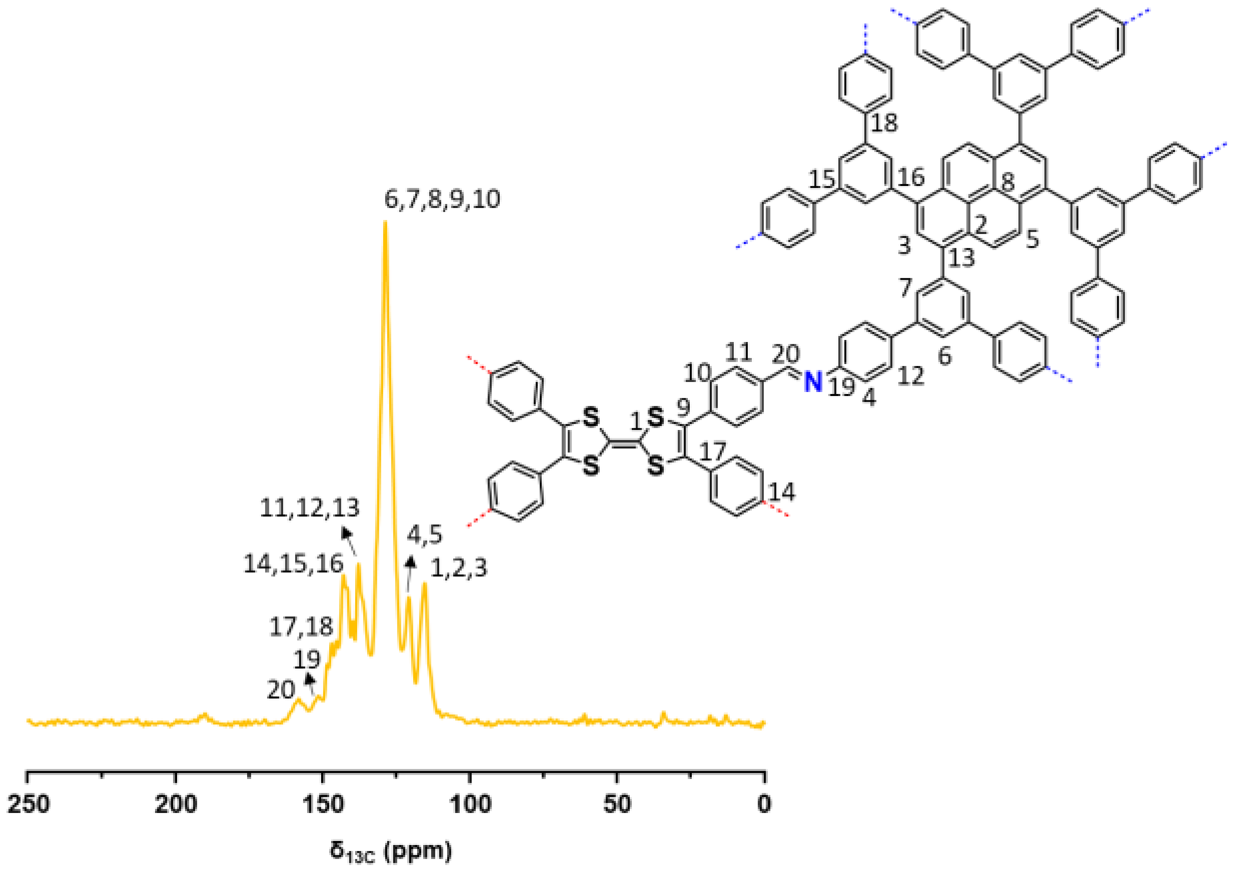

Figure S2. Solid-state ^{13}C CP-MAS NMR spectrum of TU-48.

4. Scanning electron microscopy (SEM) and Transmission electron microscopy (TEM) characterization

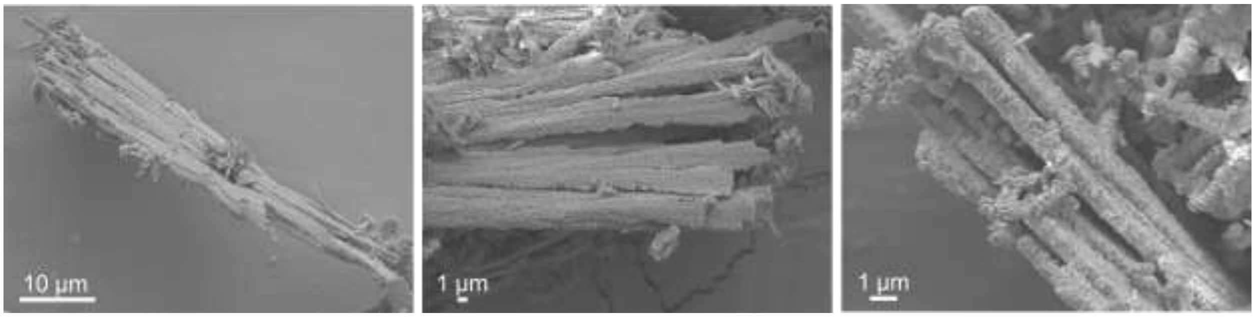

Figure S3. SEM images of TU-48.

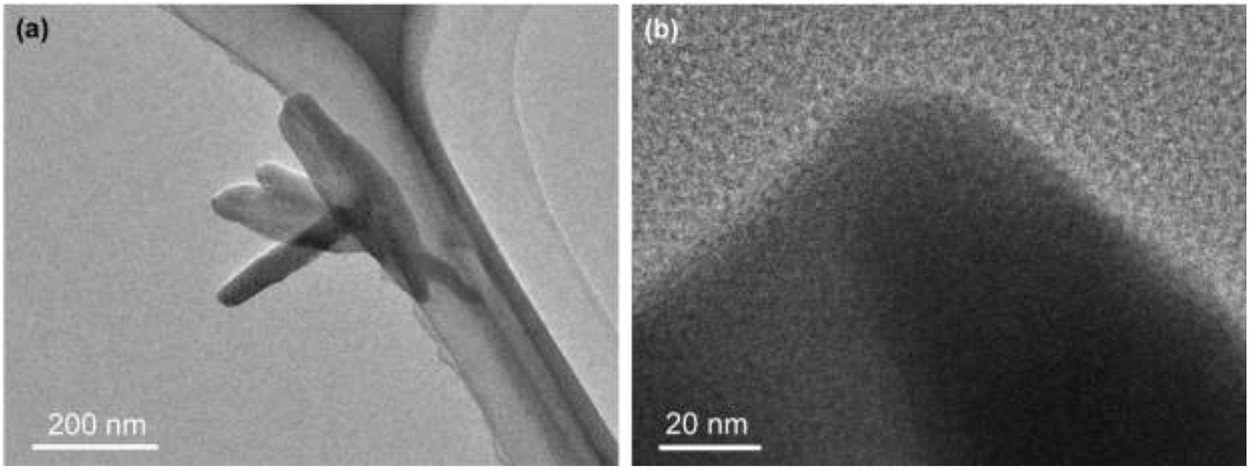

Figure S4. (a) TEM and (b) HRTEM images of TU-48.

5. Thermogravimetric analysis (TGA)

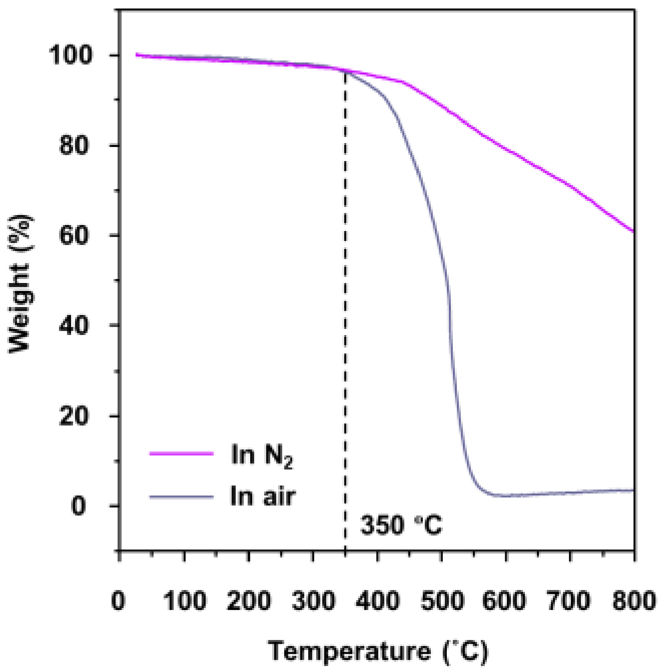

Figure S5. TGA traces of TU-48 under N_2 (purple) and air (gray) atmosphere.

6. Chemical stability analysis

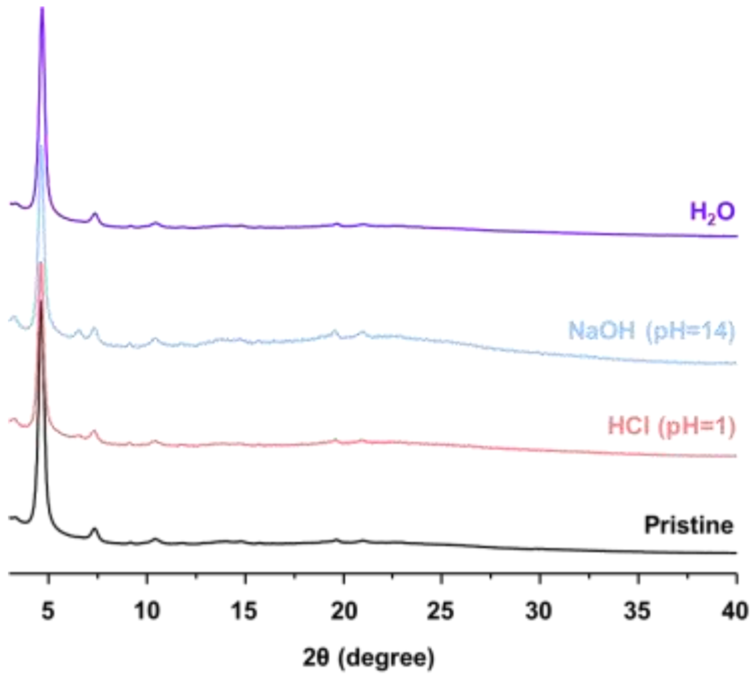

Figure S6. PXRD patterns of TU-48 after 24 hours treatment in HCl (pH=1), NaOH (pH=14) and H_2O.

7. Nitrogen sorption

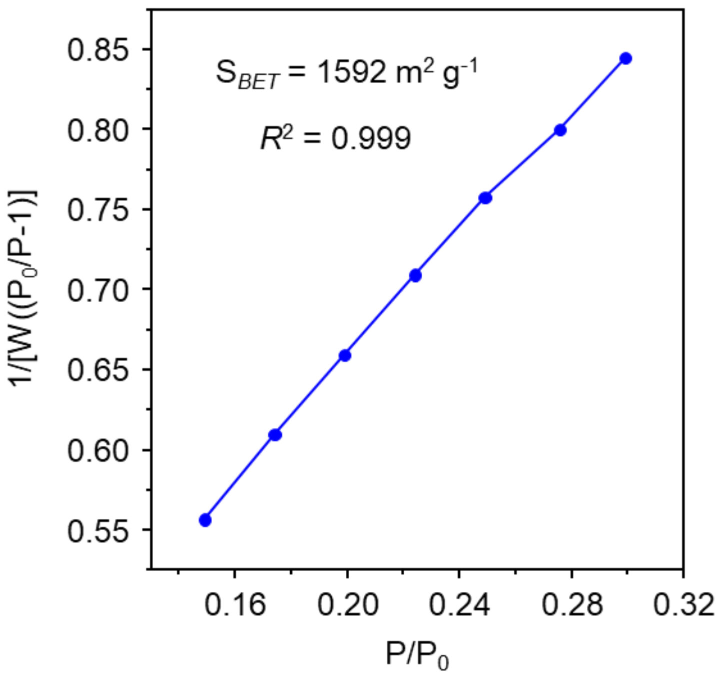

Figure S7. BET plot for TU-48 calculated from the N_2 adsorption isotherms at 77 K. S_{BET} = 1592 m^2 g^{-1}, R^2 = 0.999.

The BET surface area of TU-48 was calculated from the N_2 adsorption isotherm at 77K using the BETSI program developed by the Adsorption and Advanced Materials Lab (AAML), Department of Chemical Engineering & Biotechnology, University of Cambridge. The evaluation was performed in accordance with the protocol established by Fairen-Jimenez et al. (2022),[8] which prescribes a standardized method for BET surface area evaluation grounded in the Rouquerol criteria for accurately assessing porosity in COFs and MOFs. The complete fitting parameters are summarized below.

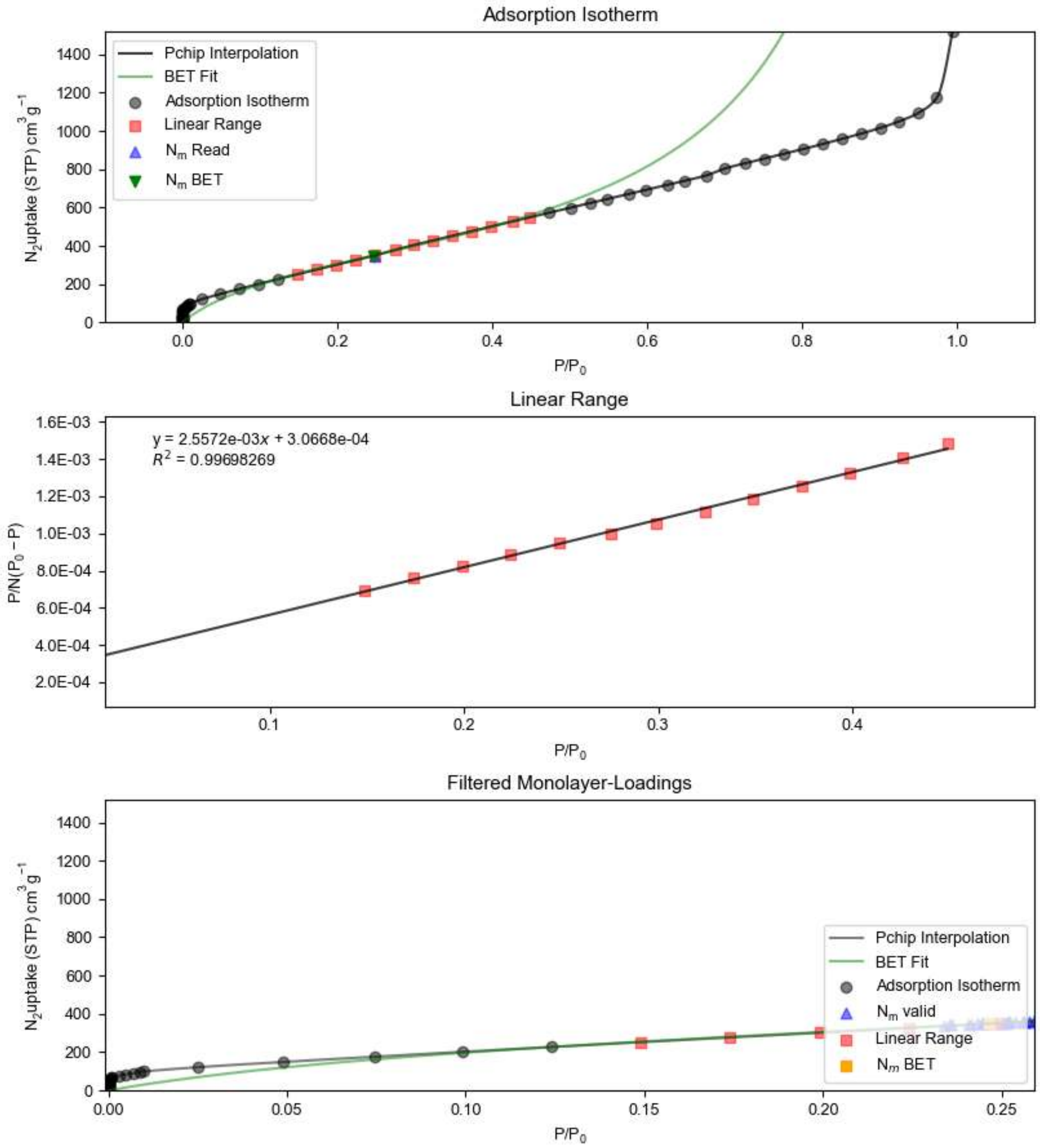

Adsorption Isotherm
Pchip Interpolation
BET Fit
Adsorption Isotherm
Linear Range
N_m Read
N_m BET
N_2uptake (STP) cm^3 g^-1
P/P_0
Linear Range
y = 2.5572e-03x + 3.0668e-04
R^2 = 0.99698269
P/N(P_0 − P)
P/P_0
Filtered Monolayer-Loadings
Pchip Interpolation
BET Fit
Adsorption Isotherm
N_m valid
Linear Range
N_m BET
N_2uptake (STP) cm^3 g^-1
P/P_0

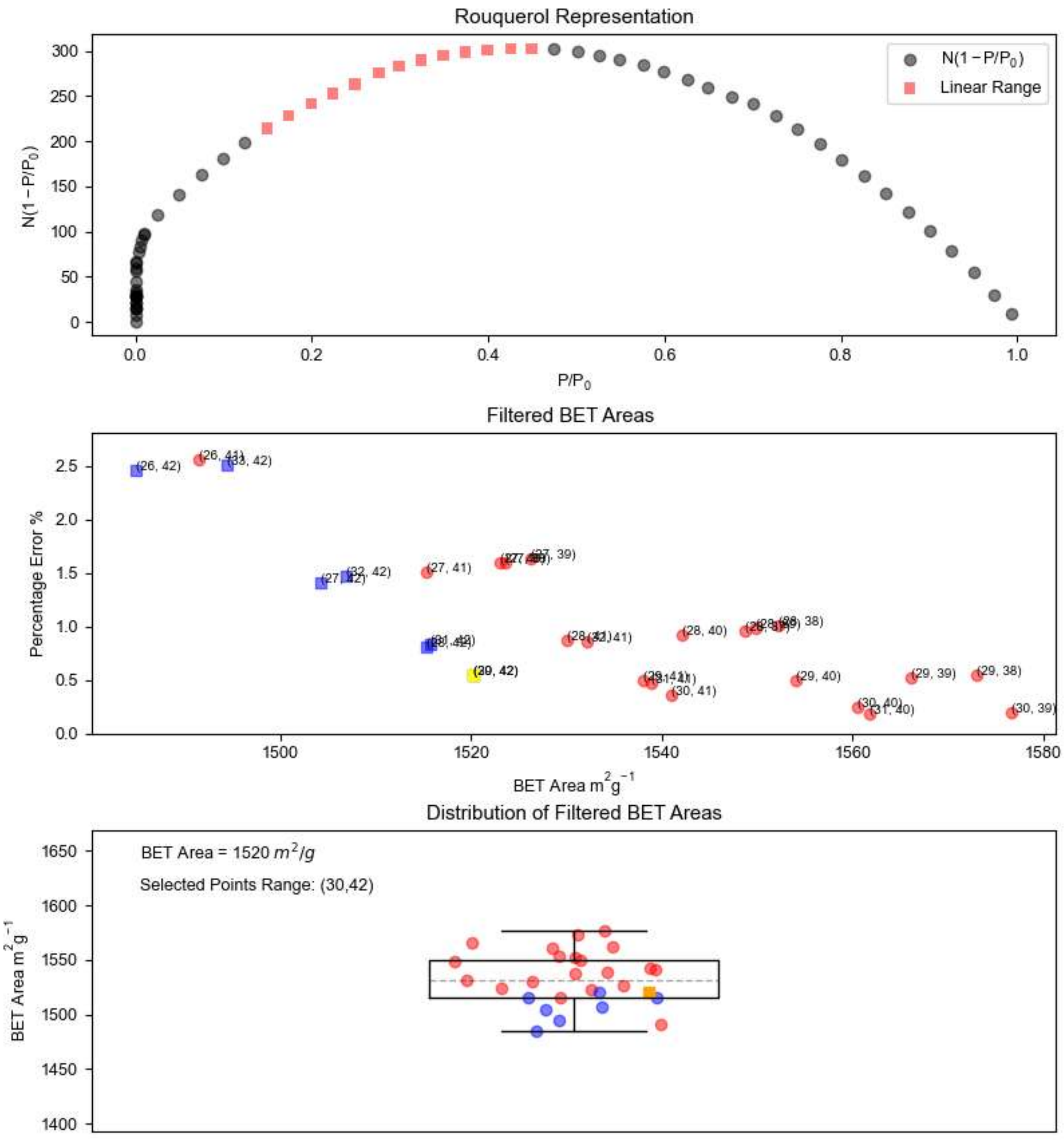

Figure S8. BETSI-derived BET surface area analysis and corresponding fitting results for TU-48.

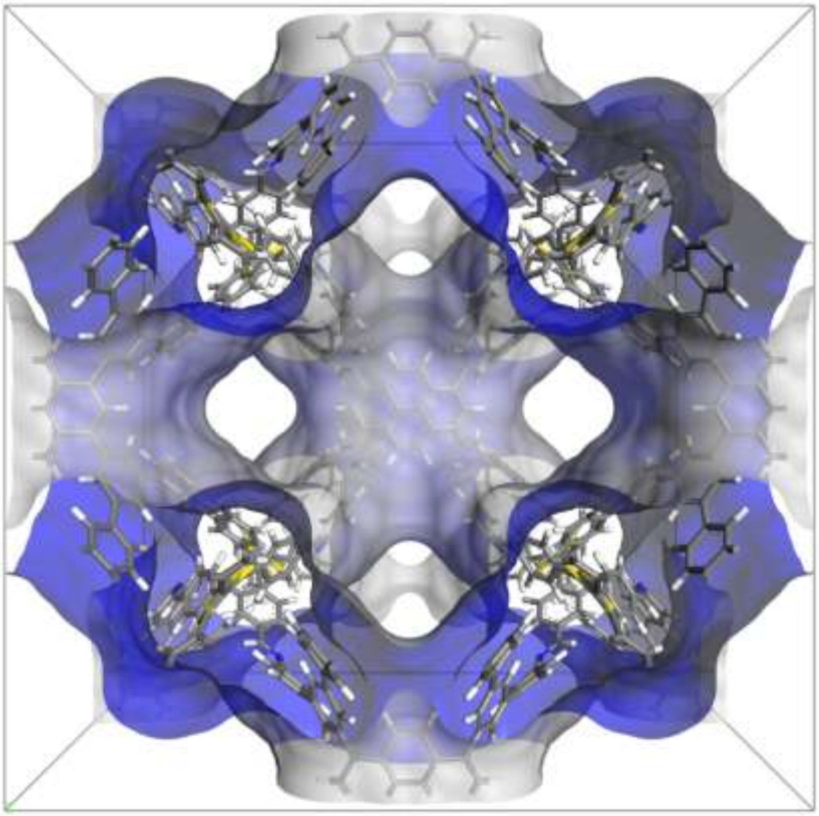

Figure S9. Pore visualizations of TU-48. Images were generated using the “Atoms, Volumes & Surfaces” tool in Accelrys Materials Studio with a probe radius of 1.82 Å.

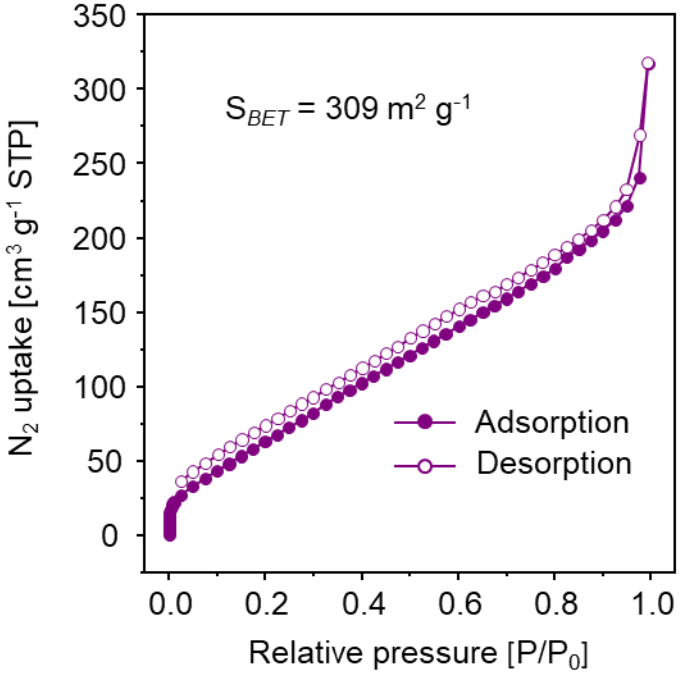

Figure S10. Nitrogen physisorption isotherms measured at 77 K for I_2-doped TU-48.

8. Diffuse reflectance spectroscopy (DRS)

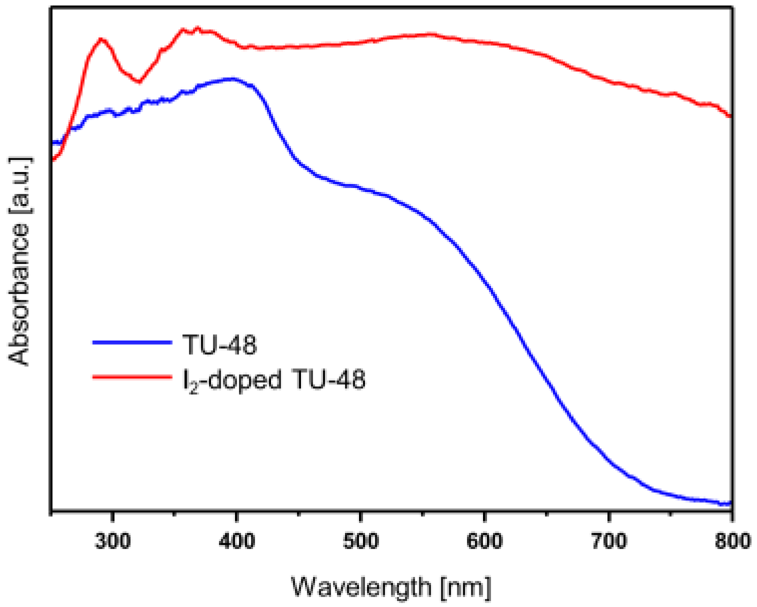

Figure S11. DRS profiles of TU-48 (blue) and its I_2-doped analogue (red).

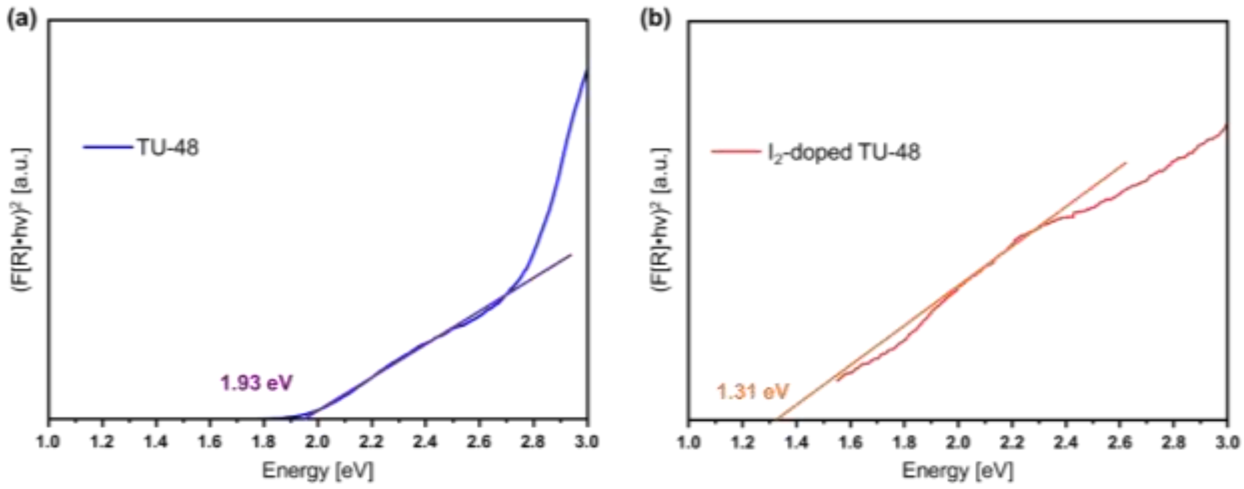

Figure S12. Tauc plots of (a) pristine TU-48 and (b) iodine-doped TU-48, constructed by plotting $(F[R]\cdot hv)^2$ (a.u.) versus photon energy (hv, eV) to estimate their optical band gaps. The extrapolation of the linear regions yields band-gap energies of 1.93 eV for pristine TU-48 and 1.31 eV for iodine-doped TU-48.

9. Energy values of HOCO and LUCO of the TU-48 COF from band structure calculations

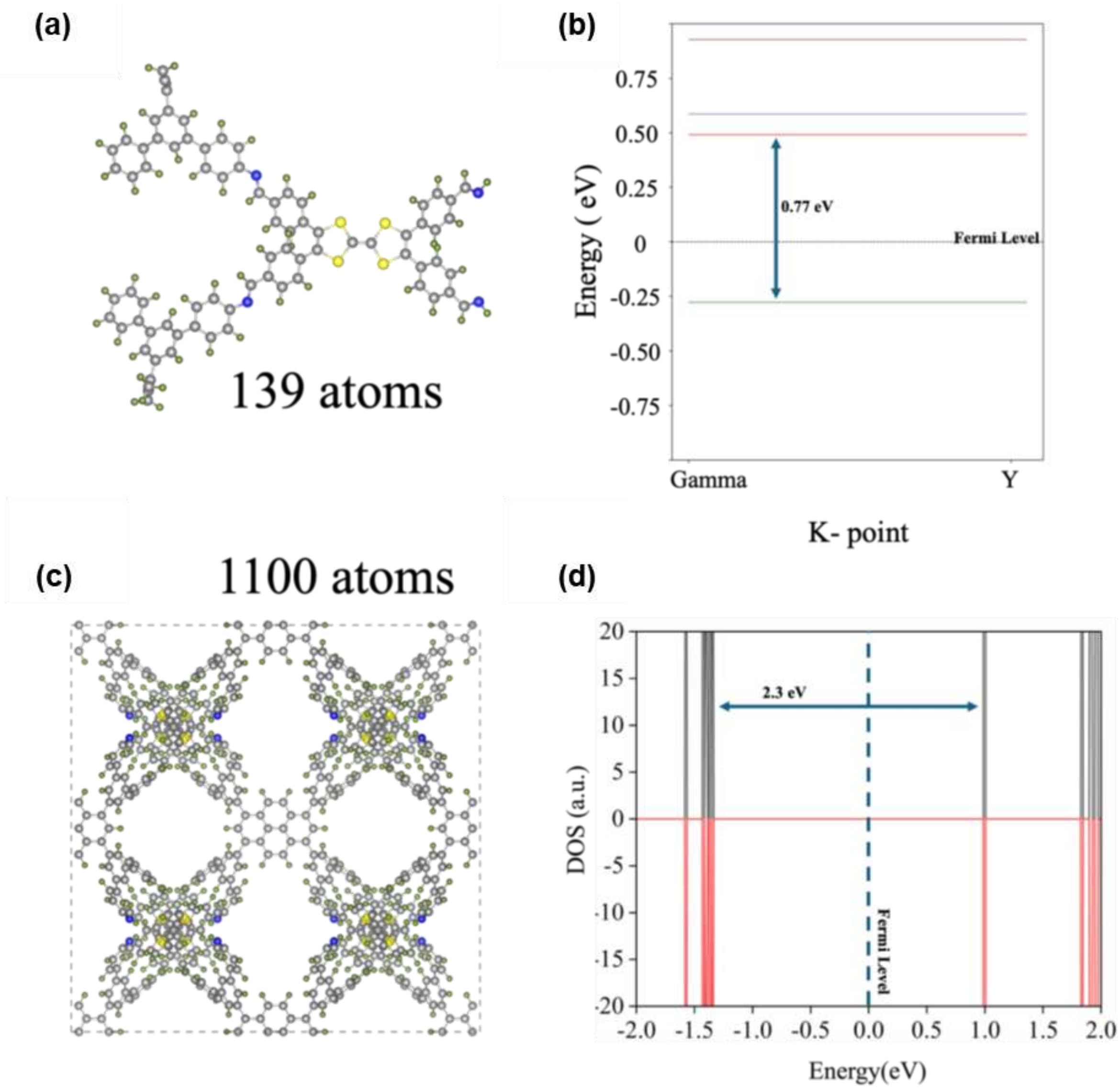

Figure S13. (a) Structure and (b) HOMO-LUMO gap of the smallest repeating unit of the COF. (c) Pristine COF structure and (d) corresponding DOS showing the HOCO-LUCO levels and band gap.

The pristine COF (Figure S13c) contains ~1100 atoms in its supercell, making a full band-structure calculation computationally prohibitive in terms of both time and memory. Therefore, we determined the band gap through an indirect but reliable approach. First, we aimed to qualitatively assess

whether the system possesses a large band gap (the experimentally reported value is ~1.9 eV). To do so, we extracted the smallest repeating unit of the COF, treated it as a “molecule in a box” (Figure S13a), and evaluated its electronic gap (Figure S13b).[9] This minimal motif exhibits a HOMO–LUMO gap of approximately 0.77 eV, with the HOMO and LUMO energies located at -3.71 eV and -2.94 eV, respectively. The corresponding Fermi level is -3.4341 eV. As expected, this value does not match the experimental band gap because the calculation is performed on an isolated repeating unit and effectively represents only one molecular chain of the COF. Nevertheless, the result clearly demonstrates that even the most elementary building block of the framework exhibits a relatively large gap.

To obtain a more accurate estimate of the electronic gap-without performing a prohibitively expensive band-structure calculation-we computed the electronic density of states (DOS) of the full COF (Figure S13d). This approach yields a band gap of ~2.3 eV, which is in closer agreement with the experimentally observed value. The highest occupied crystal orbital (HOCO) and lowest unoccupied crystal orbital (LUCO) occur at -1.3 eV and +0.99 eV, respectively. These results confirm that the extended COF indeed possesses a wide band gap, consistent with experimental trends.

10. Structural modeling and X-ray diffraction (XRD) analyses

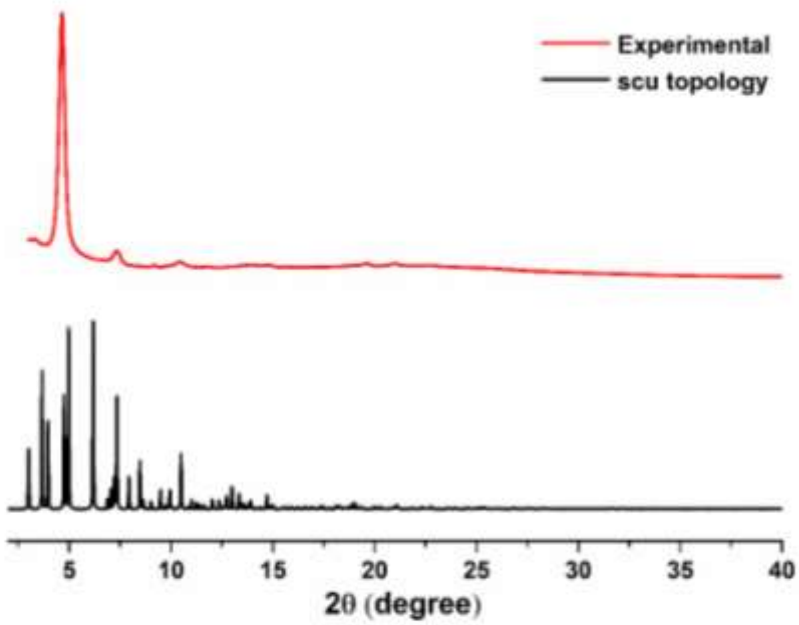

Figure S14. XRD patterns of TU-48: experimental (red) and calculated (black) based on the non-interpenetrated **scu** net with *Cmm2* space group.

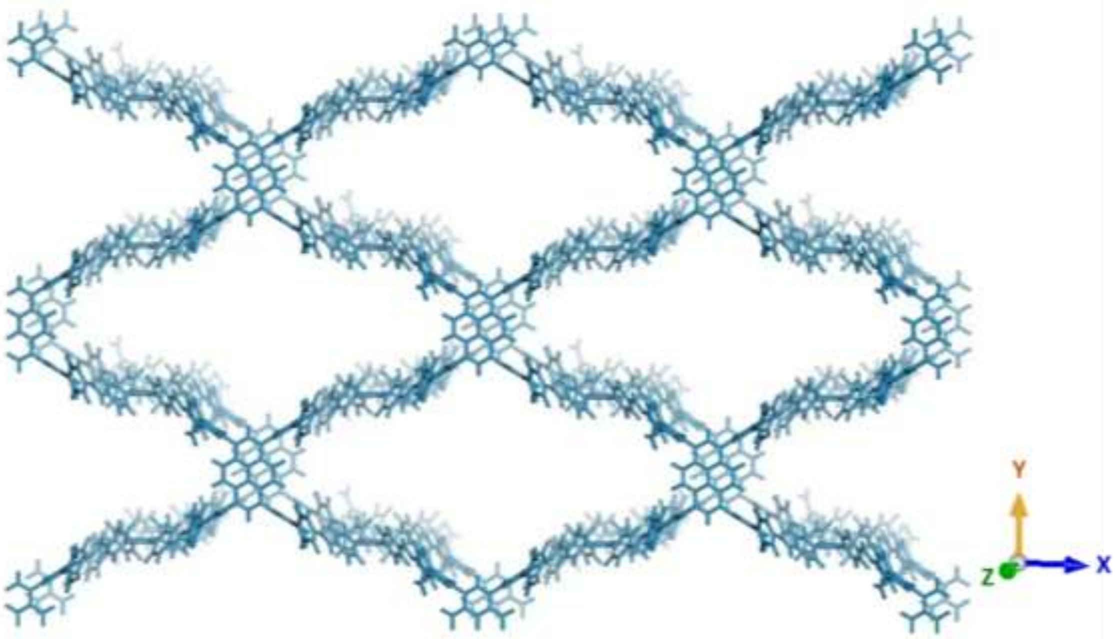

Figure S15. Structural stick model of TU-48 based on the non-interpenetrated **scu** net with *Cmm2* space group, viewed along the *z*-axis.

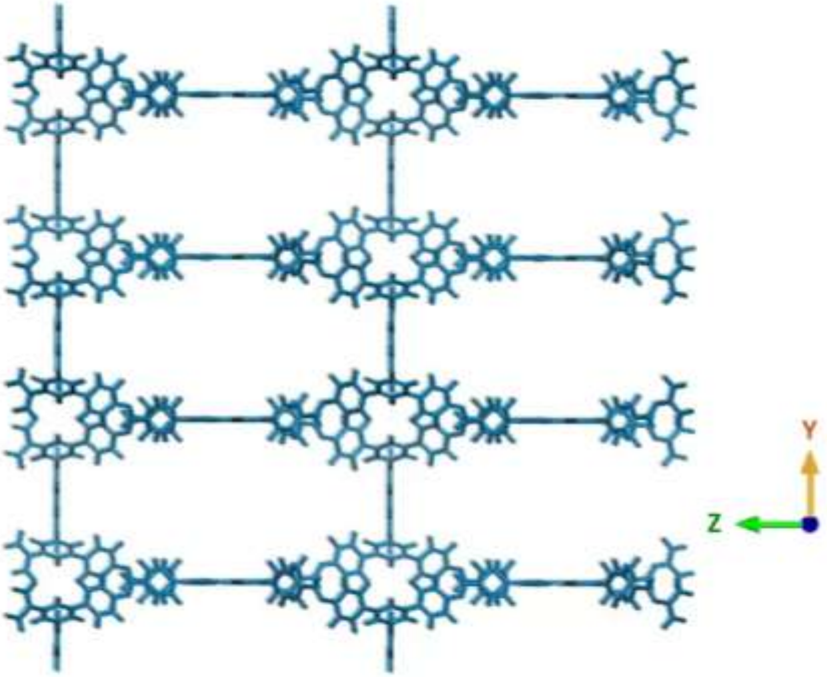

Figure S16. Structural stick model of TU-48 based on the non-interpenetrated **scu** net with *Cmm2* space group, viewed along the *x*-axis.

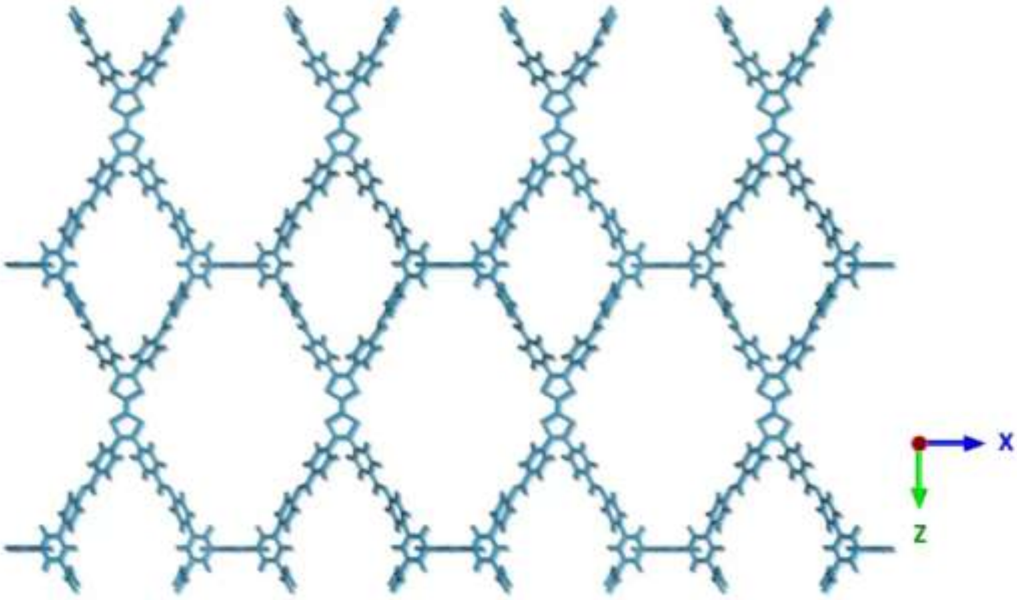

Figure S17. Structural stick model of TU-48 based on the non-interpenetrated **scu** net with *Cmm2* space group, viewed along the *y*-axis.

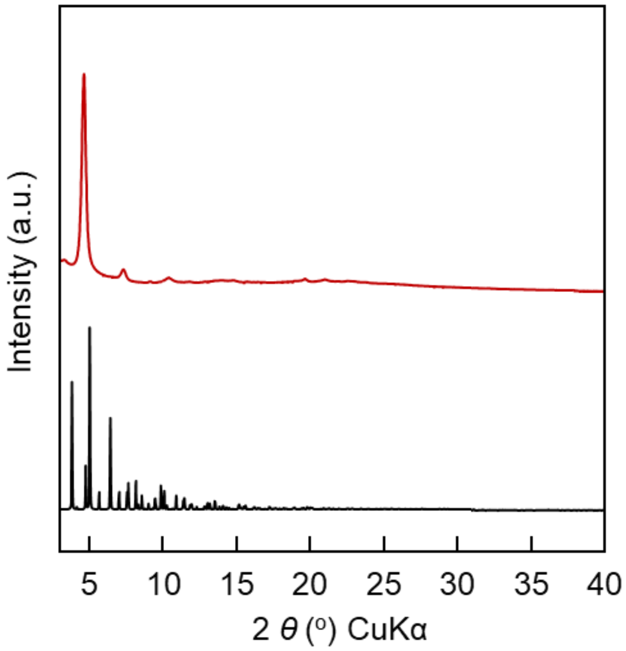

Figure S18. XRD patterns of TU-48: experimental (red) and calculated (black) based on the non-interpenetrated **scu** net with *Cmm2* space group.

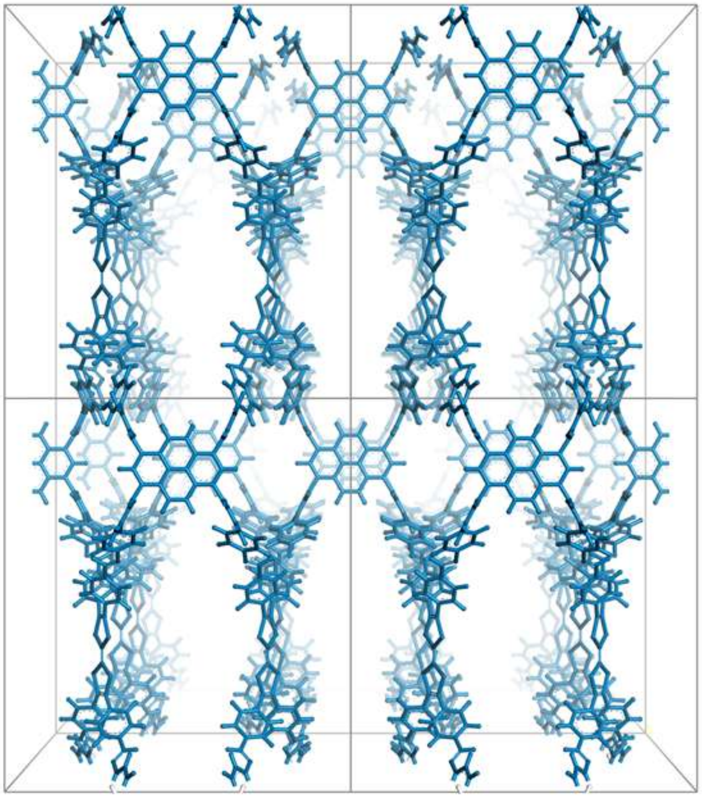

Figure S19. Structural stick model of TU-48 based on the non-interpenetrated **scu** net with *Cmm2* space group, viewed along the *y*-axis.

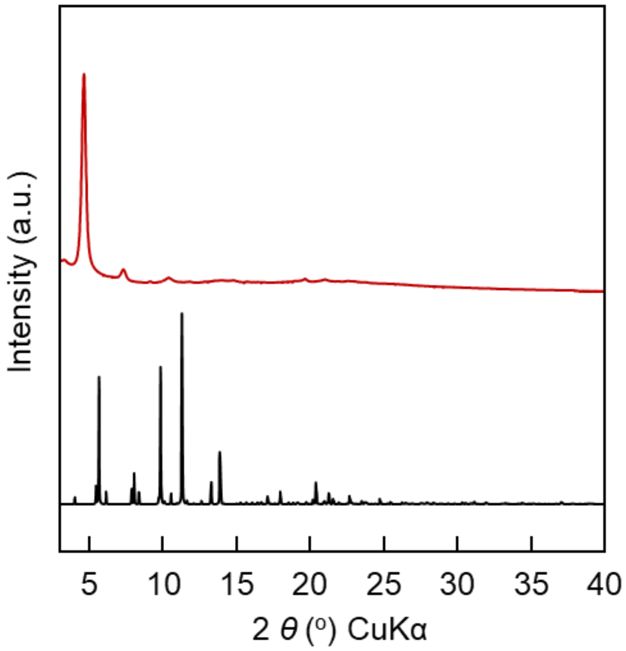

Figure S20. XRD patterns of TU-48: experimental (red) and calculated (black) based on the 2-fold interpenetrated **scu** net with *Cmc*2_1 space group.

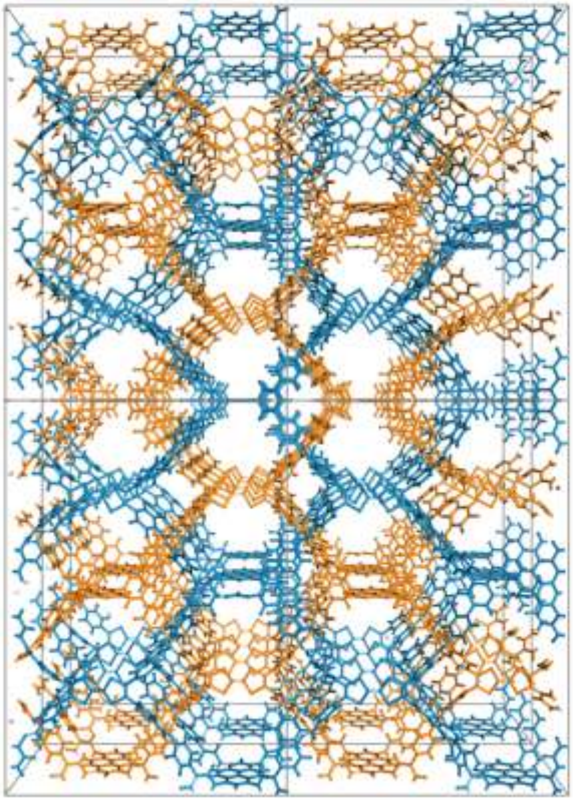

Figure S21. Structural stick model of TU-48 based on the 2-fold interpenetrated **scu** net with *Cmc*2_1 space group, viewed along the *y*-axis.

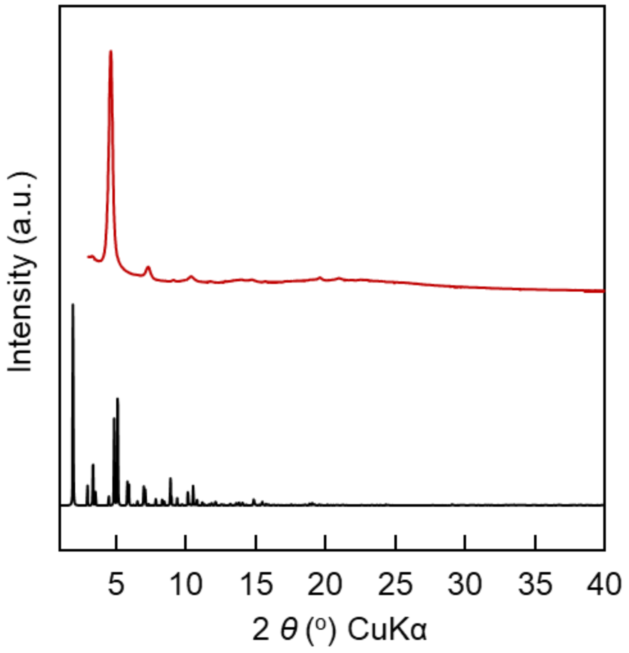

Figure S22. XRD patterns of TU-48: experimental (red) and calculated (1°-40°, black) based on the non-interpenetrated **csq** net with *P6*/*mmm* space group.

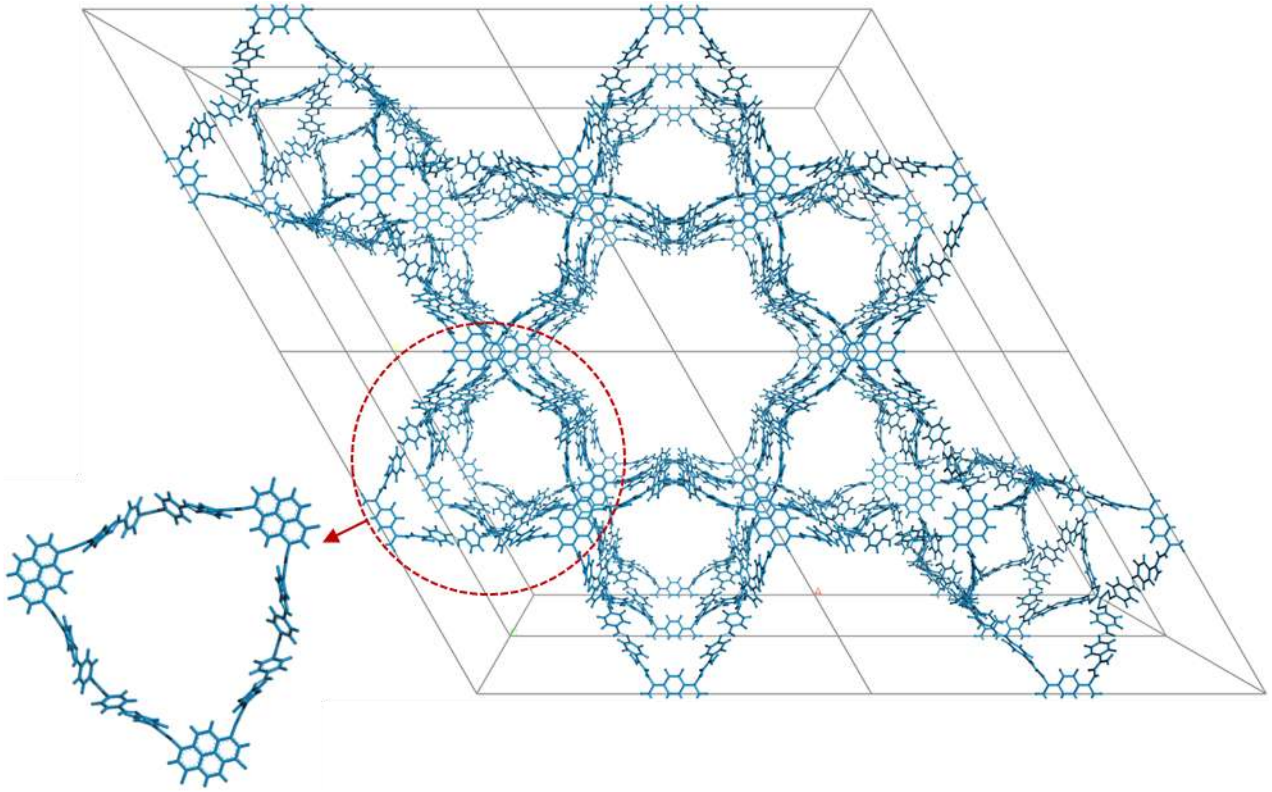

Figure S23. Structural stick model of TU-48 based on the non-interpenetrated **csq** net with *P6*/*mmm* space group, viewed along the *z*-axis.

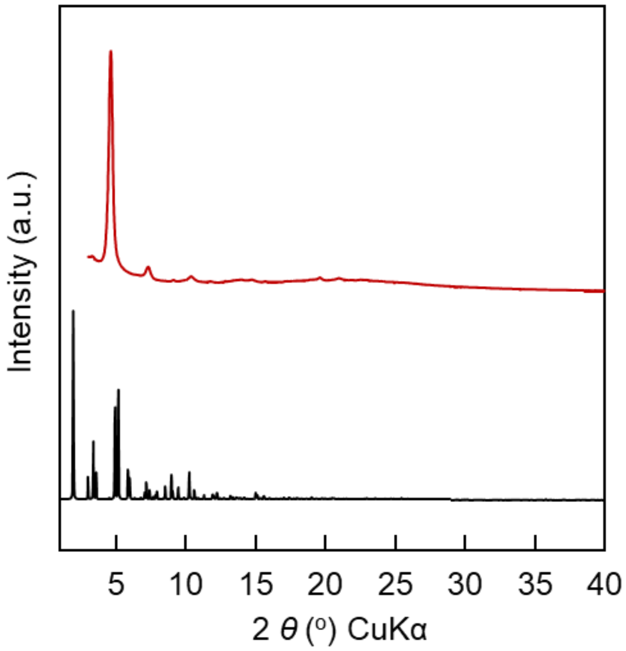

Figure S24. XRD patterns of TU-48: experimental (red) and calculated (1°-40°, black) based on the non-interpenetrated **csq** net with *P6*/*mmm* space group.

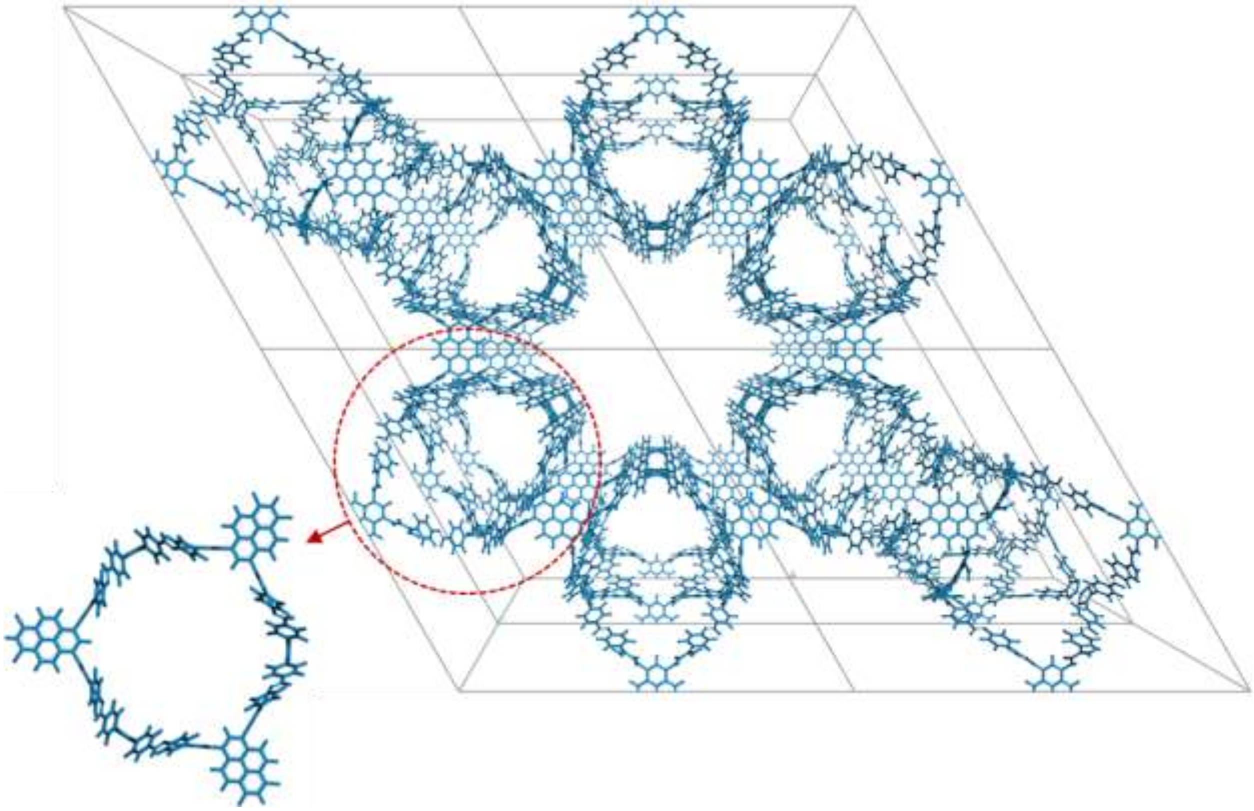

Figure S25. Structural stick model of TU-48 based on the non-interpenetrated **csq** net with *P6*/*mmm* space group, viewed along the *z*-axis.

11. Iodine doping

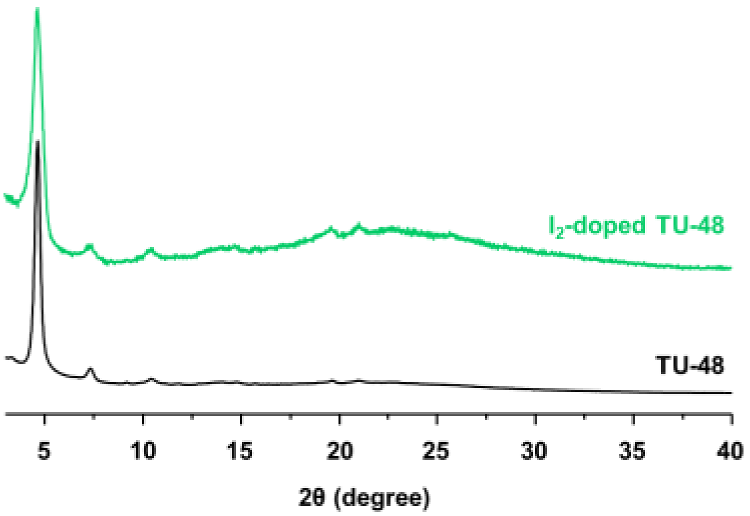

Figure S26. PXRD patterns of TU-48 in its pristine state (black) and after undergoing iodine doping for 48 hours (green).

Table S1. Iodine uptake capacity of TU-48 as a function of doping time.

Time	Before I_2 doping	After I_2 doping	I_2 uptake (wt%)	I_2/TFTTF (mol mol^{-1})
6 hours	42.9 mg	63.4 mg	47.8	2.09
12 hours	42.2 mg	82.4 mg	95.2	4.17
24 hours	42.4 mg	93.3 mg	120.0	5.26
48 hours	42.8 mg	134.4 mg	214.0	9.37

.

12. Continuous-wave X-band electron paramagnetic resonance (EPR) spectroscopy

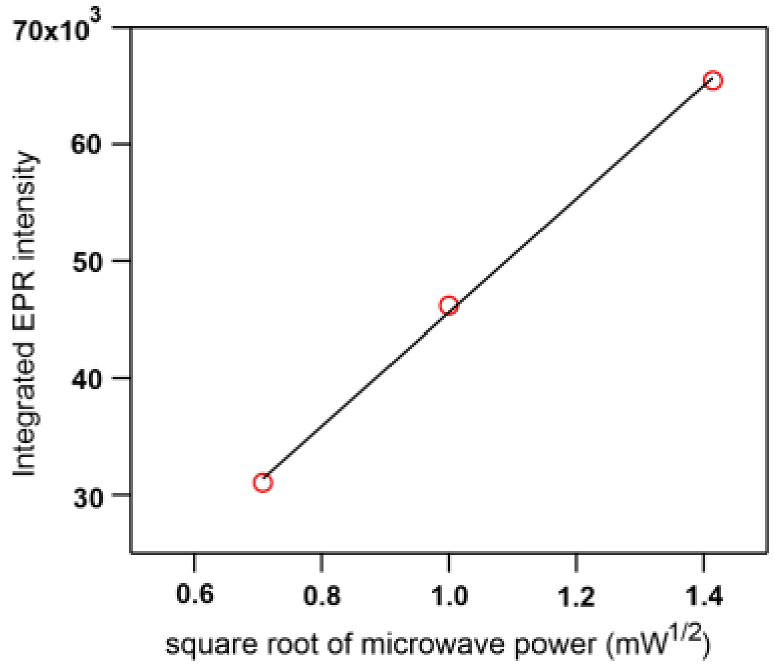

Figure S27. Power dependence of the COF EPR spectral intensity (red circles). Solid black line corresponds to linear fit (r^2 > 0.99). EPR data reported here were recorded at 1 mW microwave power.

13. Electrical conductivities of I_2-doped TU-48

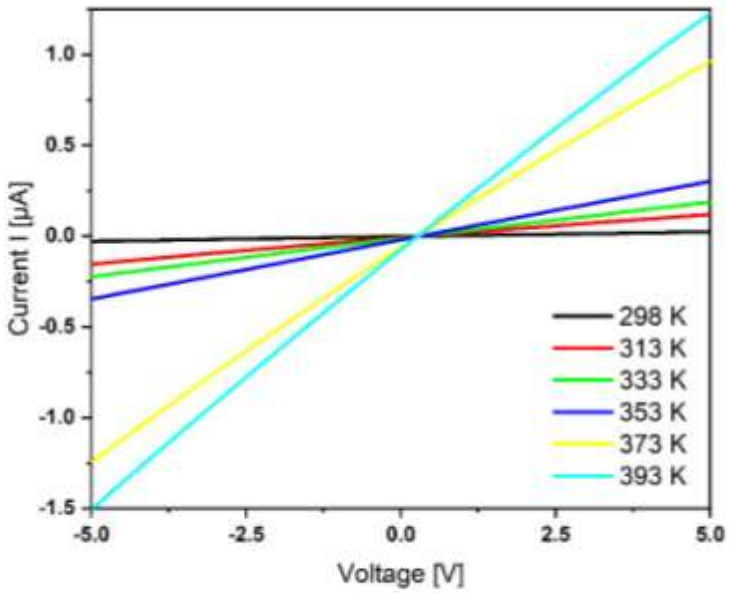

Figure S28. I-V response of TU-48 under different temperatures following I_2 oxidation for 6 hrs.

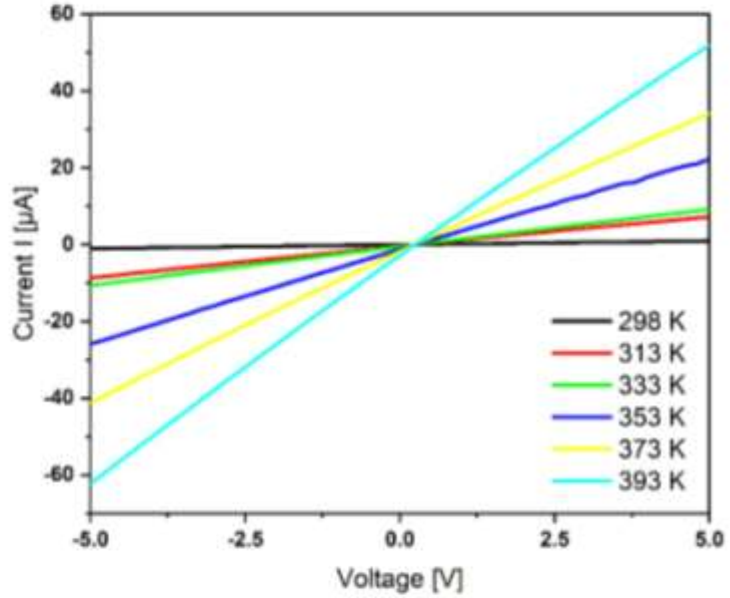

Figure S29. I-V response of TU-48 under different temperatures following I_2 oxidation for 12 hrs.

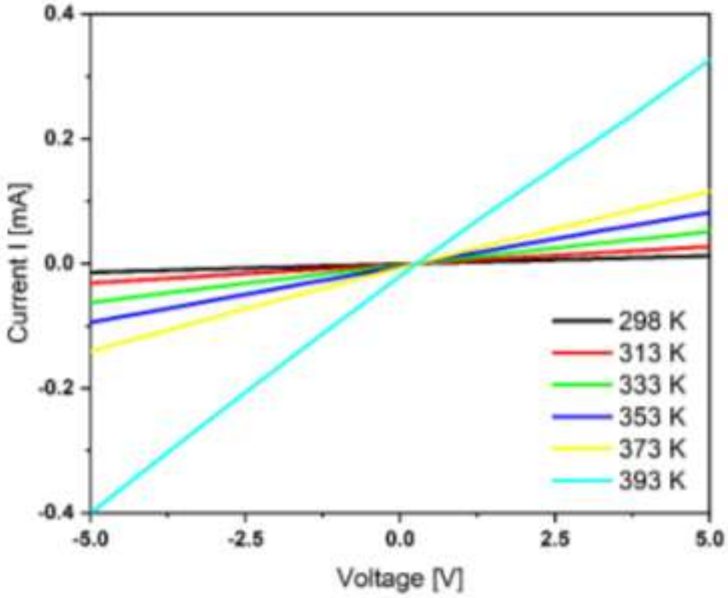

Figure S30. I-V response of TU-48 under different temperatures following I_2 oxidation for 24 hrs.

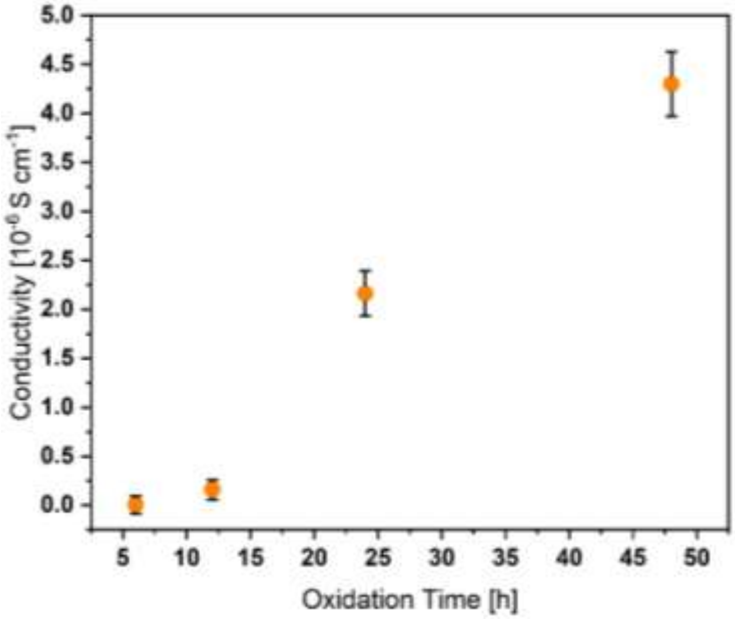

Figure S31. Impact of I_2 doping duration on the electrical conductivity of TU-48 at 298 K. The error bars represent the mean ± SD (n = 3).

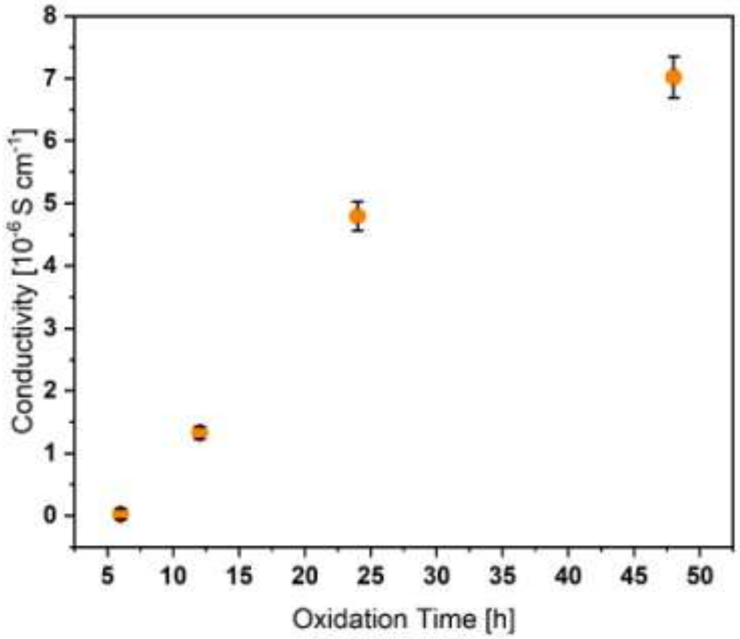

Figure S32. Impact of I_2 doping duration on the electrical conductivity of TU-48 at 313 K. The error bars represent the mean ± SD (*n* = 3).

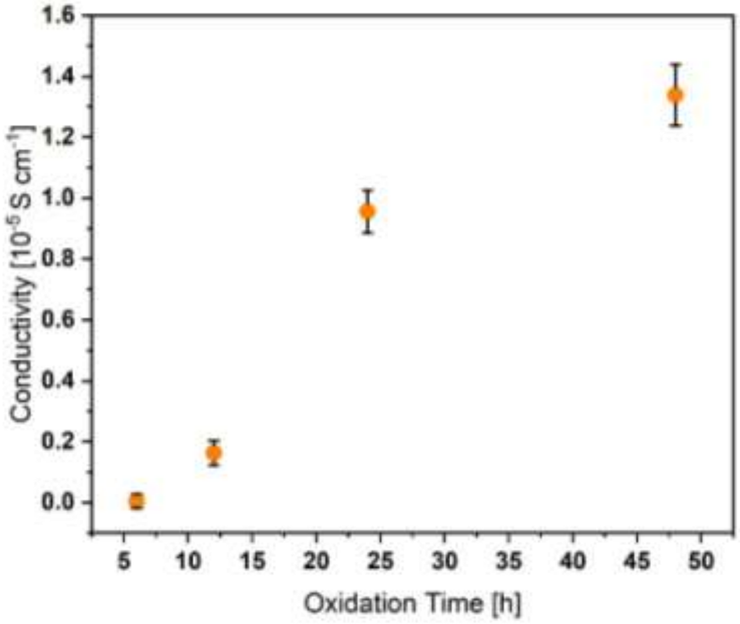

Figure S33. Impact of I_2 doping duration on the electrical conductivity of TU-48 at 333 K. The error bars represent the mean ± SD (*n* = 3).

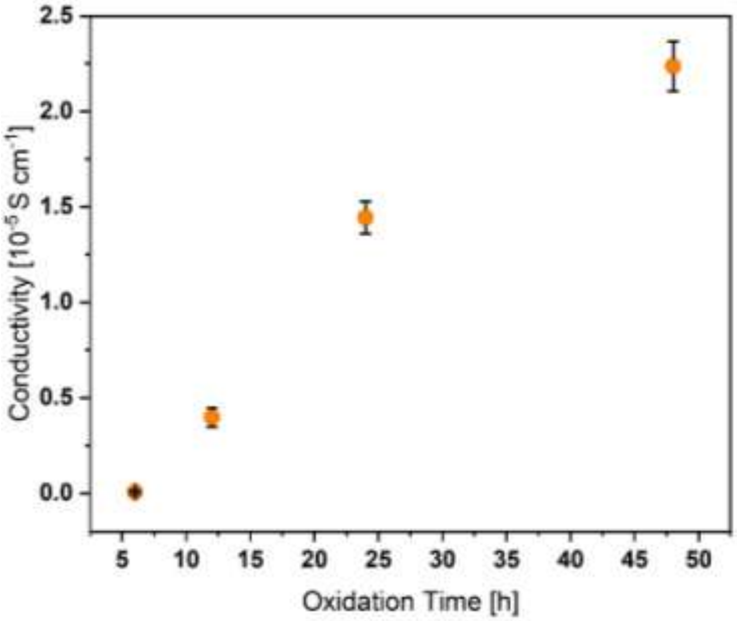

Figure S34. Impact of I_2 doping duration on the electrical conductivity of TU-48 at 353 K. The error bars represent the mean ± SD (*n* = 3).

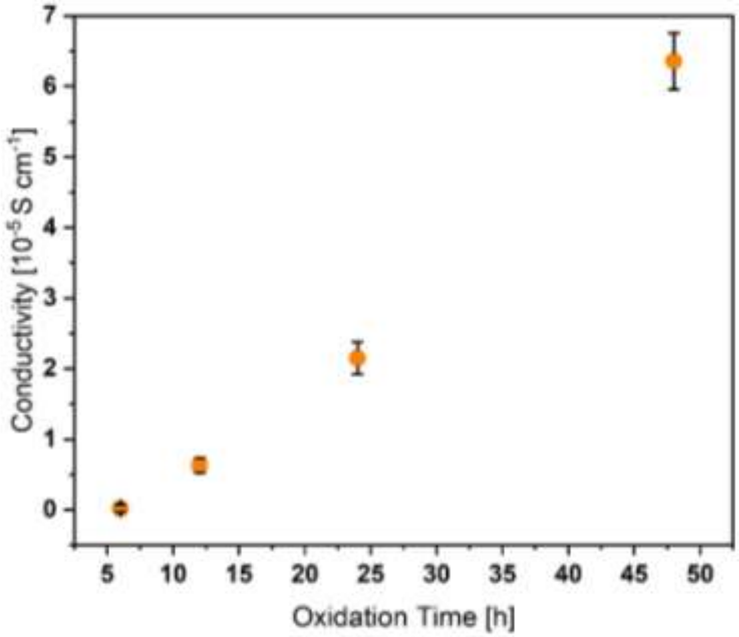

Figure S35. Impact of I_2 doping duration on the electrical conductivity of TU-48 at 373 K. The error bars represent the mean ± SD (*n* = 3).

Table S2. Conductivity performance of TU-48 under various conditions.

Oxidation time (hrs)	Temperature (K)	Conductivity (S cm^{-1})
6	298	5.1×10^{-9}
	313	2.8×10^{-8}
	333	4.0×10^{-8}
	353	6.2×10^{-8}
	373	2.2×10^{-7}
	393	2.7×10^{-7}
12	298	1.6×10^{-7}
	313	1.3×10^{-6}
	333	1.6×10^{-6}
	353	4.0×10^{-6}
	373	6.3×10^{-6}
	393	9.5×10^{-6}
24	298	2.2×10^{-6}
	313	4.8×10^{-6}
	333	9.6×10^{-6}
	353	1.4×10^{-5}
	373	2.2×10^{-5}
	393	6.1×10^{-5}
48	298	4.3×10^{-6}
	313	7.0×10^{-6}
	333	1.3×10^{-5}
	353	2.2×10^{-5}
	373	6.4×10^{-5}
	393	1.8×10^{-4}

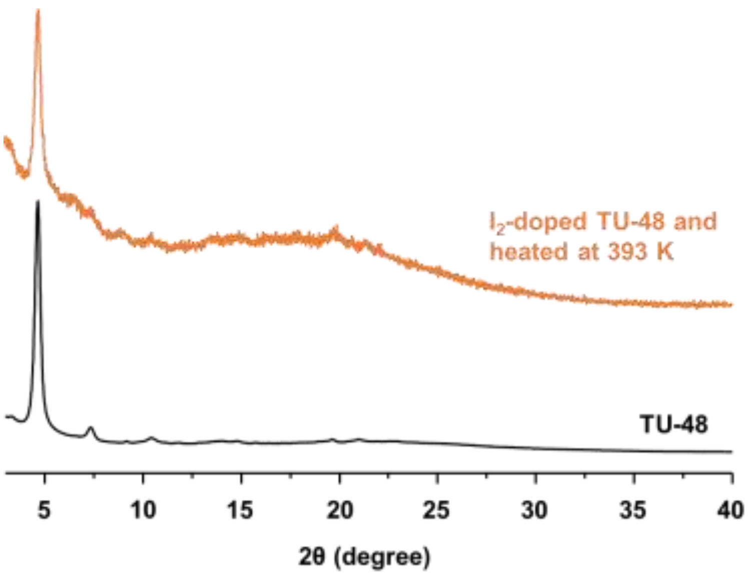

Figure S36. PXRD patterns of TU-48 in its pristine state (black) and after undergoing iodine doping for 48 hours followed by heating at 393 K (orange).

Table S3. Reported electrical conductivities of representative 2D and 3D COFs and MOFs after post-treatment (e.g., iodine doping, redox activation), compared with TU-48. Conductivity values are shown with corresponding measurement temperatures when available.

Materials	Conductivity (S cm^{-1})	Notes	Ref.
R-$TTF^{\bullet+}$-COF	3.9×10^{-3} (RT)	2D TTF-based COF + iodine	10
TTF-Ph-COF/I_2	8.50×10^{-5} (RT)	2D TTF-based COF + iodine	11
TTF-$(Ph)_2$-COF/I_2	6.98×10^{-5} (RT)	2D TTF-based COF + iodine	
TTF-BT-COF/I_2	3.98×10^{-7} (RT)	2D TTF-based COF + iodine	
Ni-TTF	6.93×10^{-5}	2D NiS_4- and TTF-based COF	12
Ni-TAP	1.57×10^{-6}	2D NiS_4-based COF	
TTF-TTF	3.83×10^{-7}	2D TTF-based COF	
I_2-doped TU-48	4.3×10^{-6} (298 K)<br>1.8×10^{-4} (393 K)	3D TTF-based COF + iodine	This work
I_2-doped TTF-COF	1.8×10^{-6} (RT)	2D TTF-based COF + iodine	13
Ni-TAPP-Co	1.18×10^{-6} (298 K)	2D NiS_4-based Co-porphyrin COF	14
TTF-TAPP-Co	9.34×10^{-7} (298 K)	2D TTF-based Co-porphyrin COF	
TTF-TAPP-2H	2.07×10^{-7} (298 K)	2D TTF-based porphyrin COF	
Ni-TAPP-2H	8.46×10^{-8} (298 K)	2D NiS_4-based porphyrin COF	
Mn_2[TTF]	9.26×10^{-7} (RT)	3D TTF-based Mn MOF	15
$Mn_2[NiS_4]$	2.88×10^{-9} (RT)	3D NiS_4-based Mn MOF	
I_x^-@TTF-8CHO-COF film	3×10^{-7}	3D TTF-based COF + iodine	16
2-Tb	1.9×10^{-7} (RT)	3D TTF-based Tb MOF + iodine	17

2-Dy	1.7×10^{-7} (RT)	3D TTF-based Dy MOF + iodine	
2-Er	1.5×10^{-7} (RT)	3D TTF-based Er MOF + iodine	
NiS_4-TAPT	4.32×10^{-8}	2D NiS_4-based COF	18
NiS_4-TAPB	4.97×10^{-8}	2D NiS_4-based COF	

14. Unit cell information and fractional atomic coordinates

Table S4. Unit cell parameters and fractional atomic coordinates for TU-48 calculated based on the 2-fold interpenetrated **scu** net with *Fmm2* space group.

Space group		*Fmm2*	
Calculated unit cell		*a* = 38.0258 Å, *b* = 37.8344 Å, *c* = 27.4576 Å, $\alpha = \beta = \gamma = 90^{o}$	
Measured unit cell		*a* = 38.0404 Å, *b* = 37.8082 Å, *c* = 27.4506 Å, $\alpha = \beta = \gamma = 90^{o}$	
Pawley refinement		R_p = 0.97%, R_{wp} = 1.44%	
Atoms	x	y	z
S1	0.27401	0.78292	0.37416
C2	0.25892	0.76531	0.43089
C3	0.23764	0.79049	0.50934
C4	0.2419	0.81297	0.54899
C5	0.27247	0.83292	0.5535
C6	0.29896	0.83039	0.51829
C7	0.29475	0.8078	0.47851
C8	0.26437	0.78715	0.47438
N9	0.30511	0.87302	0.60302
C10	0.27862	0.85187	0.5986
C11	0.30238	0.87116	0.69213
C12	0.31824	0.87825	0.73655
C13	0.34738	0.90074	0.73876
C14	0.35955	0.91732	0.69633
C15	0.34378	0.90994	0.65161
C16	0.31553	0.88641	0.64926
C17	0.38616	0.9338	0.7956
C18	0.36744	0.90327	0.78403
C19	0.26413	0.76061	0.20204
S20	0.27949	0.77776	0.25921
C21	0.30587	0.79974	0.15973
C22	0.33031	0.80633	0.12344
C23	0.33545	0.78179	0.08638
C24	0.31592	0.75039	0.08536
C25	0.29154	0.74361	0.12229
C26	0.28589	0.76868	0.1592
N27	0.35729	0.77774	0.00409
C28	0.35708	0.7933	0.046
C29	0.39831	0.82251	0.97244
C30	0.40559	0.84707	0.93617
C31	0.38882	0.8446	0.89097

C32	0.36702	0.81541	0.88132
C33	0.35975	0.79073	0.91768
C34	0.37427	0.79491	0.964
C35	0.40828	0.90574	0.86861
C36	0.38991	0.8747	0.85697
C37	0.09361	0.06471	0.33802
C38	0.13058	0.12603	0.31481
C39	0.53722	0.53229	0.35422
C40	0.51839	0.56409	0.35344
C41	0.57401	0.53214	0.34898
H42	0.2123	0.77468	0.50504
H43	0.22038	0.81504	0.57774
H44	0.32394	0.84684	0.52207
H45	0.31608	0.80609	0.44942
H46	0.25975	0.84794	0.63039
H47	0.27857	0.85288	0.69038
H48	0.30753	0.86575	0.7713
H49	0.38235	0.93683	0.69837
H50	0.35395	0.92312	0.61701
H51	0.385	0.95774	0.7704
H52	0.30191	0.8197	0.19037
H53	0.34621	0.83189	0.12396
H54	0.31999	0.73057	0.05458
H55	0.27619	0.71771	0.12245
H56	0.37453	0.81735	0.05256
H57	0.41185	0.82464	0.00959
H58	0.42523	0.86925	0.94326
H59	0.35514	0.81177	0.84352
H60	0.34198	0.76717	0.90954
H61	0.42486	0.907	0.90316
H62	0.14606	0.15086	0.30503
H63	0.53301	0.59041	0.35281
C64	0.59197	0.5	0.34686
C65	0.51874	0.5	0.35635
H66	0.37978	0.5	0.34137
C67	0.75	0.75	0.84089
C68	0.75	0.75	0.79254

Table S5. Unit cell parameters and fractional atomic coordinates for TU-48 calculated based on the non-interpenetrated **scu** net with *Cmm2* space group shown in Figures S10-S12.

Space group		*Cmm2*	
Calculated unit cell		a = 44.6299 Å, b = 28.6242 Å, c = 29.3654 Å, $\alpha = \beta = \gamma = 90^o$	
Atoms	x	y	z
C1	0.05494	0.1268	-0.12643
C2	0.12396	0.21721	-0.03407
C3	0.11309	0.17219	-0.04358
C4	0.11845	0.13603	-0.0123
C5	0.13454	0.14468	-0.97237
C6	0.13966	0.226	-0.99375
C7	0.14493	0.18967	-0.96245
N8	0.16187	0.19668	-0.92147
C9	0.16391	0.23579	-0.89922
C10	0.17863	0.27695	-0.82796
C11	0.19564	0.28016	-0.78808
C12	0.21708	0.24595	-0.77786
C13	0.22097	0.20803	-0.80757
C14	0.20367	0.20443	-0.84705
C15	0.18235	0.23893	-0.85761
S16	0.21546	0.24918	-0.68196
C17	0.23498	0.24933	-0.73568
C18	0.10772	0.1478	-0.25011
C19	0.10762	0.17974	-0.21377
C20	0.12154	0.22336	-0.21969
C21	0.13571	0.23451	-0.26078
C22	0.12187	0.15892	-0.29111
C23	0.13632	0.20192	-0.29676
N24	0.15164	0.21021	-0.3391
C25	0.16886	0.24534	-0.34913
C26	0.20461	0.28382	-0.40223
C27	0.22066	0.28512	-0.44306
C28	0.2168	0.24986	-0.47581
C29	0.19576	0.21441	-0.46792
C30	0.17971	0.21306	-0.42708
C31	0.18428	0.24748	-0.39359
S32	0.21551	0.24766	-0.57181
C33	0.23495	0.24936	-0.518
C34	0.05456	-0.02432	-0.12491
C35	0.59389	0.66685	0.83054

C36	0.56994	0.63704	0.91421
C37	0.59676	0.66264	0.91355
C38	0.56686	0.64169	0.83193
C39	0.60849	0.67768	0.87165
C40	0.52752	0.54954	0.8751
C41	0.52722	0.59892	0.87441
H42	0.12018	0.24559	-0.05777
H43	0.11067	0.10089	-0.0192
H44	0.1388	0.11628	-0.94889
H45	0.14817	0.26083	-0.98769
H46	0.15107	0.26603	-0.90975
H47	0.16232	0.30392	-0.83542
H48	0.19234	0.30959	-0.76544
H49	0.23723	0.18105	-0.79994
H50	0.2071	0.17481	-0.86939
H51	0.09753	0.11367	-0.24646
H52	0.12141	0.24896	-0.19253
H53	0.14601	0.26849	-0.26378
H54	0.1221	0.1335	-0.31842
H55	0.17337	0.27258	-0.3246
H56	0.20839	0.31087	-0.37708
H57	0.23641	0.31329	-0.44889
H58	0.19236	0.18706	-0.49288
H59	0.16407	0.18471	-0.4215
H60	0.076	-0.04201	-0.12516
H61	0.56069	0.62508	0.94629
H62	0.55513	0.63331	0.80067
H63	0.62972	0.69605	0.871
C64	0.25	0.25	-0.64983
C65	0.25	0.25	-0.60394
C66	0.5	0.37722	-0.12608
C67	0.5	0.52478	0.87533

15. Supplementary references

(1) Gong, C.; Wang, H.; Sheng, G.; Wang, X.; Xu, X.; Wang, J.; Miao, X.; Liu, Y.; Zhang, Y.; Dai, F.; Chen, L.; Li, N.; Xu, G.; Jia, J.; Zhu, Y.; Peng, Y. Synthesis and Visualization of Entangled 3D Covalent Organic Frameworks with High-Valency Stereoscopic Molecular Nodes for Gas Separation. *Angew. Chem., Int. Ed.* **2022**, *61*, e202204899.

(2) Mitamura, Y.; Yorimitsu, H.; Oshima, K.; Osuka, A. Straightforward access to aryl-substituted tetrathiafulvalenes by palladium-catalysed direct C–H arylation and their photophysical and electrochemical properties. *Chem. Sci.* **2011**, *2*, 2017-2021.

(3) Giannozzi, P.; Baseggio, O.; Bonfà, P.; Brunato, D.; Car, R.; Carnimeo, I.; Cavazzoni, C.; de Gironcoli, S.; Delugas, P.; Ferrari Ruffino, F.; Ferretti, A.; Marzari, N.; Timrov, I.; Urru, A.; Baroni, S. Quantum ESPRESSO toward the Exascale. *J. Chem. Phys.* **2020**, *152*, 154105.

(4) Giannozzi, P.; Baroni, S.; Bonini, N.; Calandra, M.; Car, R.; Cavazzoni, C.; Ceresoli, D.; Chiarotti, G. L.; Cococcioni, M.; Dabo, I.; Corso, A. D.; de Gironcoli, S.; Fabris, S.; Fratesi, G.; Gebauer, R.; Gerstmann, U.; Gougoussis, C.; Kokalj, A.; Lazzeri, M.; Martin-Samos, L.; Marzari, N.; Mauri, F.; Mazzarello, R.; Paolini, S.; Pasquarello, A.; Paulatto, L.; Sbraccia, C.; Scandolo, S.; Sclauzero, G.; Seitsonen, A. P.; Smogunov, A.; Umari, P.; Wentzcovitch, R. M. QUANTUM ESPRESSO: A Modular and Open-Source Software Project for Quantum Simulations of Materials. *J. Phys. Condens. Matter* **2009**, *21*, 395502.

(5) Giannozzi, P.; Andreussi, O.; Brumme, T.; Bunau, O.; Nardelli, M. B.; Calandra, M.; Car, R.; Cavazzoni, C.; Ceresoli, D.; Cococcioni, M.; Colonna, N.; Carnimeo, I.; Corso, A. D.; de Gironcoli, S.; Delugas, P.; DiStasio, R. A.; Ferretti, A.; Floris, A.; Fratesi, G.; Fugallo, G.; Gebauer, R.; Gerstmann, U.; Giustino, F.; Gorni, T.; Jia, J.; Kawamura, M.; Ko, H.-Y.; Kokalj, A.; Küçükbenli, E.; Lazzeri, M.; Marsili, M.; Marzari, N.; Mauri, F.; Nguyen, N. L.; Nguyen, H.-V.; Otero-de-la-Roza, A.; Paulatto, L.; Poncé, S.; Rocca, D.; Sabatini, R.; Santra, B.; Schlipf, M.; Seitsonen, A. P.; Smogunov, A.; Timrov, I.; Thonhauser, T.; Umari, P.; Vast, N.; Wu, X.; Baroni, S. Advanced Capabilities for Materials Modelling with Quantum ESPRESSO. *J. Phys. Condens. Matter* **2017**, *29*, 465901.

(6) Perdew, J. P.; Burke, K.; Ernzerhof, M. Generalized Gradient Approximation Made Simple. *Phys. Rev. Lett.* **1996**, *77*, 3865.

(7) Monkhorst, H. J.; Pack, J. D. Special Points for Brillouin-Zone Integrations. *Phys. Rev. B* **1976**, *13*, 5188.

(8) Osterrieth, J. W. M.; Rampersad, J.; Madden, D.; Rampal, N.; Skoric, L.; Connolly, B.; Allendorf, M. D.; Stavila, V.; Snider, J. L.; Ameloot, R.; Marreiros, J.; Ania, C.; Azevedo, D.; Vilarrasa-Garcia, E.; Santos, B. F.; Bu, X.-H.; Chang, Z.; Bunzen, H.; Champness, N. R.; Griffin, S. L.; Chen, B.; Lin, R.-B.; Coasne, B.; Cohen, S.; Moreton, J. C.; Colón, Y. J.; Chen,

L.; Clowes, R.; Coudert, F.-X.; Cui, Y.; Hou, B.; D'Alessandro, D. M.; Doheny, P. W.; Dincă, M.; Sun, C.; Doonan, C.; Huxley, M. T.; Evans, J. D.; Falcaro, P.; Ricco, R.; Farha, O.; Idrees, K. B.; Islamoglu, T.; Feng, P.; Yang, H.; Forgan, R. S.; Bara, D.; Furukawa, S.; Sanchez, E.; Gascon, J.; Telalović, S.; Ghosh, S. K.; Mukherjee, S.; Hill, M. R.; Sadiq, M. M.; Horcajada, P.; Salcedo-Abraira, P.; Kaneko, K.; Kukobat, R.; Kenvin, J.; Keskin, S.; Kitagawa, S.; Otake, K.-i.; Lively, R. P.; DeWitt, S. J. A.; Llewellyn, P.; Lotsch, B. V.; Emmerling, S. T.; Pütz, A. M.; Martí-Gastaldo, C.; Padial, N. M.; García-Martínez, J.; Linares, N.; Maspoch, D.; Suárez del Pino, J. A.; Moghadam, P.; Oktavian, R.; Morris, R. E.; Wheatley, P. S.; Navarro, J.; Petit, C.; Danaci, D.; Rosseinsky, M. J.; Katsoulidis, A. P.; Schröder, M.; Han, X.; Yang, S.; Serre, C.; Mouchaham, G.; Sholl, D. S.; Thyagarajan, R.; Siderius, D.; Snurr, R. Q.; Goncalves, R. B.; Telfer, S.; Lee, S. J.; Ting, V. P.; Rowlandson, J. L.; Uemura, T.; Iiyuka, T.; van der Veen, M. A.; Rega, D.; Van Speybroeck, V.; Rogge, S. M. J.; Lamaire, A.; Walton, K. S.; Bingel, L. W.; Wuttke, S.; Andreo, J.; Yaghi, O.; Zhang, B.; Yavuz, C. T.; Nguyen, T. S.; Zamora, F.; Montoro, C.; Zhou, H.; Kirchon, A.; Fairen-Jimenez, D. *Adv. Mater.* **2022**, *34*, 2201502.

(9) Ding, C.; Xie, X.; Chen, L.; Troisi, A. Intuitive and Efficient Approach to Determine the Band Structure of Covalent Organic Frameworks from Their Chemical Constituents. *J. Chem. Theory Comput.* **2024**, *20*, 1252-1262.

(10) Cao, S.; Partovi-Azar, P.; Yang, J.; Xie, D.; Held, T.; Marcozzi, G.; E. McPeak, J.; Zhang, W.; Zhang, X.; Osenberg, M.; Kochovski, Z.; Li, C.; Sebastiani, D.; Schmidt, J.; Exner, M.; Manke, I.; Thomas, A.; Wang, W.; Lu, Y. A Radical-Cationic Covalent Organic Framework to Accelerate Polysulfide Conversion for Long-Durable Lithium−Sulfur Batteries. *J. Am. Chem. Soc.* **2025**, *147*, 31073-31084.

(11) Valente, G.; Dantas, R.; Ferreira, P.; Grieco, R.; Patil, N.; Guillem-Navajas, A.; Rodríguez-San Miguel, D.; Zamora, F.; Guntermann, R.; Bein, T.; Rocha, J.; Helena Braga, M.; Strutyński, K.; Melle-Franco, M.; Marcilla, R.; Souto, M. Tetrathiafulvalene-based covalent organic frameworks as high-voltage organic cathodes for lithium batteries. *J. Mater. Chem. A,* **2024**, *12*, 24156-24164.

(12) Li, Y.-Y.; Wei, T.; Liu, C.; Zhang, Z.; Wu, L.-F.; Ding, M.; Yuan, S.; Zhu, J.; Zuo, J.-L. Integrating Tetrathiafulvalene and Nickel-Bis(dithiolene) Units into Donor-Acceptor Covalent Organic Frameworks for Stable and Efficient Photothermal Conversion. *Chem. Eur. J.* **2023**, *29*, e202301048.

(13) Ding, H.; Li, Y.; Hu, H.; Sun, Y.; Wang, J.; Wang, C.; Wang, C.; Zhang, G.; Wang, B.; Xu, W.; Zhang, D. A Tetrathiafulvalene-Based Electroactive Covalent Organic Framework. *Chem. Eur. J.* **2014**, *20*, 14614-14618.

(14) Ke, S.-W. Li, W.; Gu, Y.; Su, J.; Liu, Y.; Yuan, S.; Zuo, J.-L.; Ma, J.; He, P. Covalent organic frameworks with Ni-Bis(dithiolene)and Co-porphyrin units as bifunctional catalysts for Li-O_2

batteries. *Sci. Adv.* **2023**, *9*, eadf2398.

(15) Zhou, X.-C.; Liu, C.; Su, J.; Liu, Y.-F.; Mu, Z.; Sun, Y.; Yang, Z.-M.; Yuan, S.; Ding, M.; Zuo, J.-L. Redox-Active Mixed-Linker Metal–Organic Frameworks with Switchable Semiconductive Characteristics for Tailorable Chemiresistive Sensing. *Angew. Chem., Int. Ed.* **2023**, *62*, e202211850.

(16) Ma, T.-R.; Ge, F.; Ke, S.-W.; Lv, S.; Yang, Z.-M.; Zhou, X.-C.; Liu, C.; Wu, X.-J.; Yuan, S.; Zuo, J.-L. Accessible Tetrathiafulvalene Moieties in a 3D Covalent Organic Framework for Enhanced Near-Infrared Photo-Thermal Conversion and Photo-Electrical Response. *Small* **2024**, *20*, 2308013.

(17) Su, J.; Yuan, S.; Li, J.; Wang, H.-Y.; Ge, J.-Y.; F. Drake, H.; F. Leong, C.; Yu, F.; M. D'Alessandro, D.; Kurmoo, M.; Zuo, J.-L.; Zhou, H.-C. Rare-Earth Metal Tetrathiafulvalene Carboxylate Frameworks as Redox-Switchable Single-Molecule Magnets. *Chem. Eur. J.* 2021, *27*, 622-627.

(18) Lv, S.; Ma, X.; Ke, S.; Wang, Y.; Ma, T.; Yuan, S.; Jin, Z.; Zuo, J.-L. Metal-Coordinated Covalent Organic Frameworks as Advanced Bifunctional Hosts for Both Sulfur Cathodes and Lithium Anodes in Lithium−Sulfur Batteries. *J. Am. Chem. Soc.* **2024**, *146*, 9385-9394.
